# Convergent functional, white-matter microstructural, and cerebral perfusion abnormalities in overweight and obese adults: a multimodal MRI study

**DOI:** 10.3389/fnut.2026.1917977

**Published:** 2026-09-03

**Authors:** Hua Yin, Yuanyuan Yi, Yuhang Yu, Lingqianhui Liu, Xiaohui Wu, Ruisui Huang, Lifeng Huang, Wenqi Qin, Mi Qin, Wen Yang, Ke Ding

**Affiliations:** 1 Department of Radiology, The Third Affiliated Hospital of Guangxi Medical University, Nanning, Guangxi, China; 2 Department of Radiology, Nanxishan Hospital of Guangxi Zhuang Autonomous Region, Guilin, Guangxi, China

**Keywords:** arterial spin labeling (ASL), body mass index (BMI), diffusion tensor imaging (DTI), obesity, resting-state functional MRI (RS-fMRI)

## Abstract

**Background:**

Obesity affects the brain, but most neuroimaging studies examine a single modality, leaving it unclear whether obesity-related functional, microstructural, and perfusion abnormalities co-localize. We combined three modalities across the normal-weight–overweight–obese spectrum.

**Methods:**

Of 80 adults enrolled, 10 were excluded (sleep, incomplete acquisition, artifacts), leaving 70 (normal-weight, *n* = 27; overweight, *n* = 21; obese, *n* = 22), who did not differ in age, sex, or education. All underwent resting-state functional MRI, diffusion tensor imaging, and 3D arterial spin labeling at 3.0 T, yielding amplitude of low-frequency fluctuation (ALFF), regional homogeneity (ReHo), functional connectivity (FC), fractional anisotropy (FA), mean (MD), axial, and radial diffusivity (RD), and cerebral blood flow (CBF). Group differences were assessed with covariate-adjusted models; diffusion metrics were tested across all 48 atlas regions with false-discovery-rate (FDR) correction, and body mass index (BMI) relationships by whole-sample regression without prior region selection. Spatial convergence was tested by minimum-statistic conjunction and random-displacement permutation.

**Results:**

Obese participants showed lower ALFF than controls in the cerebellar posterior lobe (vermis, left lobule VIII, right Crus II) and left superior frontal gyrus, higher ALFF in the bilateral parahippocampal gyri, lower ReHo in the left superior frontal gyrus, and lower ReHo than overweight participants in the left middle cingulate gyrus. No regional diffusion difference survived FDR correction, but BMI predicted FA, MD, and RD in the right inferior cerebellar peduncle in whole-sample regression (all *q* < 0.015), the only associations to survive. CBF was reduced in the cerebellar posterior lobe and increased in the left inferior frontal and right postcentral gyri. ALFF–CBF overlap there exceeded chance at every threshold, reaching 43.1-fold at *P* < 0.001, confirmed by conjunction and permutation (*P* = 0.0002–0.0008); peak-level in the left lobule VIII and right Crus II, but atlas-level only at the vermis. FC showed no group differences.

**Conclusion:**

Overweight and obesity are associated with spatially convergent functional, microstructural, and perfusion abnormalities preferentially involving cerebellar and cortico-limbic regions, scaling continuously with BMI in the right inferior cerebellar peduncle. Because cognition was assessed only by the MMSE, these roles suggest hypotheses rather than conclusions about cognition.

## 1 Introduction

Obesity, characterized by abnormal or excessive fat accumulation that adversely affects health, has become one of the most pressing public-health problems worldwide (1). Its prevalence has risen steeply over recent decades, and China now carries a very large absolute burden of overweight and obesity (2). Beyond its well-established association with cardiometabolic disease (3), obesity is increasingly linked to accelerated brain aging and to neuropsychiatric and neurodegenerative conditions (4, 5). The brain is therefore now regarded as an important target organ in obesity, and characterizing obesity-related brain alterations has become a priority for early identification and intervention.

Body mass index (BMI) remains the most widely used clinical index of adiposity, and Chinese criteria define overweight as 24.0 ≤ BMI < 28.0 kg/m^2^ and obesity as BMI ≥ 28.0 kg/m^2^ (6, 7). Although early models attributed obesity primarily to an imbalance between energy intake and expenditure, energy-homeostasis frameworks alone cannot account for the complexity of human eating behavior in food-abundant environments, where hedonic, reward-driven eating frequently overrides homeostatic need (8). This has shifted attention toward central mechanisms—particularly cortico-limbic circuits subserving reward, executive control, and emotion regulation—and obesity has been associated with a relative imbalance between heightened reward responsiveness and weakened executive control (8, 9). Consistent with this view, resting-state functional studies have repeatedly demonstrated altered spontaneous activity and connectivity in reward- and control-related regions in obesity (10, 11).

Magnetic resonance imaging (MRI) provides non-invasive, complementary windows onto these processes. Resting-state functional MRI (rs-fMRI) infers spontaneous neural activity from blood-oxygen-level-dependent signals: ALFF indexes the intensity of local spontaneous activity (12, 13), ReHo reflects local synchrony (14), and seed-based FC captures inter-regional coupling (15); such metrics reveal altered spontaneous activity and connectivity in obesity (10, 11). Diffusion tensor imaging (DTI) is the principal non-invasive method for assessing white-matter microstructure; FA, MD, AD, and RD provide complementary information on overall fiber integrity, total diffusivity, and the axonal and myelin compartments, respectively (16). Reduced white-matter integrity, including lower FA in tracts such as the corpus callosum, has been reported in obesity, and white-matter changes have been discussed in relation to obesity-associated cognitive differences (17, 18, 19, 16). Arterial spin labeling (ASL) non-invasively quantifies cerebral blood flow (CBF), an early indicator of neural dysfunction, and has been applied to characterize cerebral perfusion in obesity and related conditions (20–24).

Despite this progress, most prior studies have examined a single imaging modality, making it difficult to determine whether obesity-related functional, microstructural, and perfusion abnormalities co-localize within the same regions. Moreover, DTI studies have largely emphasized FA and MD, with AD and RD—which help distinguish axonal from myelin involvement—reported far less often (16). Few studies have integrated function, structure, and perfusion within a single, demographically matched cohort, or examined changes across the full normal-weight–overweight–obese spectrum.

Accordingly, we combined rs-fMRI, DTI, and 3D-ASL in a single cohort of normal-weight, overweight, and obese adults matched for age, sex, and education, in order to (i) characterize functional, white-matter microstructural, and perfusion alterations; (ii) identify regions in which abnormalities converge across modalities; and (iii) relate these indices to BMI. We hypothesized that obesity would be associated with spatially overlapping multimodal abnormalities preferentially involving cerebellar and cortico-limbic regions. Because the affected regions are known to participate in executive, affective, and sensorimotor processing, we interpret our findings in terms of these regional functions rather than measured cognition; the cognitive relevance of the observed changes is considered separately in light of the study’s assessment limitations.

## 2 Materials and methods

### 2.1 Participants

This study was approved by the Ethics Committee of the Third Affiliated Hospital of Guangxi Medical University (approval No. Y2021097) and was conducted in accordance with the Declaration of Helsinki. All participants provided written informed consent after the study purpose, procedures, and potential risks had been explained. Between October 2024 and January 2026, 50 overweight/obese patients (overweight, *n* = 25; obese, *n* = 25) and 30 normal-weight volunteers were recruited from the outpatient and inpatient services and the surrounding community. Ten participants were subsequently excluded because they had fallen asleep during scanning, because acquisition was incomplete, or because of image artifacts (three normal-weight, four overweight, and three obese), leaving 70 participants with complete and analyzable rs-fMRI, DTI, and ASL data (normal-weight, *n* = 27; overweight, *n* = 21; obese, *n* = 22). All analyses reported below, including the demographic and clinical characteristics in Table 1, are based on these 70 participants. Within the obese group, participants were additionally classified by obesity grade, but the resulting subgroups were too small for separate analysis and were therefore pooled.

**TABLE 1:** Demographic and clinical characteristics of the three groups.

Variable	Normal-weight (*n* = 27)	Overweight (*n* = 21)	Obese (*n* = 22)	Statistic	*P*
Sex, male/female (n)	13/14	11/10	13/9	χ^2^ = 0.585	0.746
Age, median (IQR), years	32.0 (27.5–45.5)	42.0 (27.0–53.0)	29.5 (26.0–40.5)	*H* = 2.426	0.297
Educational level, n (%)				*H* = 0.553	0.758
Primary school or below	1 (3.7)	2 (9.5)	1 (4.5)		
Secondary school	8 (29.6)	8 (38.1)	6 (27.3)		
Junior college	4 (14.8)	1 (4.8)	6 (27.3)		
Bachelor’s degree or above	14 (51.9)	10 (47.6)	9 (40.9)		
MMSE, median (IQR)	30 (30–30)	30 (30–30)	30 (30–30)	*H* = 4.554	0.103
BMI, median (IQR), kg/m^2^	21.5 (20.1–22.2)	25.8 (24.5–26.7)	29.9 (28.7–34.6)	*H* = 61.050	< 0.001

Data are n or median (interquartile range). Group comparisons used the chi-square test (sex) or the Kruskal–Wallis test. Educational categories were comparable across groups (*H* = 0.553, *P* = 0.758). MMSE, Mini-Mental State Examination; BMI, body mass index; NW, normal-weight; OW, overweight; OB, obese.

Patients were eligible if they met Chinese criteria for overweight/obesity (BMI ≥ 24 kg/m^2^), were right-handed, had complete and analyzable rs-fMRI, DTI, and ASL data, and had completed the Mini-Mental State Examination (MMSE) before scanning. Normal-weight controls were required to have a BMI of 18.5–24 kg/m^2^ and to meet the same remaining criteria. Exclusion criteria comprised psychiatric disease; severe organic disease or functional impairment of the heart, lung, brain, or other major organs; a history of alcohol or substance abuse; inability to complete scanning (e.g., body habitus exceeding the bore, claustrophobia); contraindications to MRI (e.g., pacemakers or ferromagnetic implants); image artifacts precluding analysis; and overweight/obesity secondary to medication or other identifiable causes. General clinical information (sex, age, height, weight, educational level) and the MMSE were recorded for all participants. To minimize circadian variability, all scans were performed between 19:00 and 20:00.

### 2.2 Image acquisition

All examinations were performed on a 3.0-T MRI system (uMR790; United Imaging Healthcare) with a transmit head coil and a 32-channel phased-array head receive coil. Foam padding and earplugs were used to limit head motion and acoustic noise; participants were instructed to lie still with eyes closed, remain awake, and avoid structured thought, and were questioned immediately after scanning to exclude those who had fallen asleep. rs-fMRI used a gradient-echo echo-planar sequence [repetition time (TR) 2,000 ms, echo time (TE) 30 ms, field of view (FOV) 220 × 220 mm, slice thickness 3.5 mm, no gap, 36 slices, matrix 64 × 64, flip angle 80°, 180 volumes]. DTI used TR 9941 ms, TE 78.6 ms, slice thickness 2 mm, no gap, 75 slices, and matrix 128 × 128, with diffusion encoding along 32 non-collinear directions at b = 1,000 s/mm^2^ in addition to a *b* = 0 image (2 averages, NEX = 2), FOV 256 × 256 mm. 3D-ASL was acquired axially (TR/TE 5000/13.86 ms, slice thickness 4 mm, 34 slices, FOV 224 × 224 mm, post-labeling delay 1,800 ms, labeling duration 1,800 ms). A single post-labeling delay was used.

### 2.3 rs-fMRI processing

rs-fMRI data were processed in DPARSF/DPABI on MATLAB R2022b (25, 26). After conversion from DICOM to NIfTI, the first 10 volumes were discarded; the remaining volumes underwent slice-timing correction and realignment, with participants showing translation > 3 mm or rotation > 3° excluded. Images were spatially normalized to Montreal Neurological Institute (MNI) space and resampled to 3-mm isotropic voxels, detrended, and regressed against nuisance covariates (white-matter and cerebrospinal-fluid signals and 24 head-motion parameters), followed by temporal band-pass filtering (0.01–0.1 Hz). Spatial smoothing with a 6-mm full-width-at-half-maximum (FWHM) Gaussian kernel was applied for all metrics except ReHo. ALFF and ReHo were then computed for each participant. Seed-based FC was examined using composite seeds defined by the regions showing the most robust ALFF/ReHo group differences (the left superior frontal gyrus and the right cerebellar Crus II).

### 2.4 DTI processing

DTI data were processed using MRtrix3 (27), FSL (28), and ANTs. After format conversion, images underwent denoising (dwidenoise), Gibbs-ringing removal, motion and eddy-current correction, and N4 bias-field correction; non-brain tissue was removed and a brain mask was generated. Diffusion tensors were fitted (FSL dtifit) to obtain FA, MD, AD, and RD maps. T1-weighted images were skull-stripped and used to register diffusion data to standard space; the Johns Hopkins University (JHU) white-matter atlas was transformed into diffusion space, and mean values were extracted within each atlas-defined region of interest. The JHU ICBM-DTI-81 parcellation comprises 48 regions, all of which were analyzed.

### 2.5 ASL processing

ASL data were processed in MATLAB (25). Control and label images were subtracted and averaged, and CBF was computed together with a proton-density-weighted reference image (29). CBF maps were normalized to a perfusion template in MNI space (SPM), restricted to brain tissue, and normalized to the whole-brain mean gray-matter CBF to reduce inter-individual differences in global perfusion, before smoothing with a 6-mm FWHM Gaussian kernel.

### 2.6 Statistical analysis

Demographic and clinical variables were compared among groups using the chi-square test and the Kruskal–Wallis test, as appropriate. For ALFF and ReHo, voxel-wise two-sample *t*-tests were performed with sex and age as covariates, and the resulting statistical maps were corrected using Gaussian random field (GRF) theory (voxel-level *P* < 0.005, cluster-level *P* < 0.05). For CBF, voxel-wise two-sample *t*-tests with sex and age as covariates were corrected with GRF (voxel-level *P* < 0.001, cluster-level *P* < 0.05). Significant clusters were localized using the Anatomical Automatic Labeling atlas. To determine whether the functional and perfusion findings overlapped more than would be expected by chance, the ALFF and CBF t-maps for the obese-versus-normal-weight comparison were additionally compared at thresholds common to both modalities. Because the two maps had identical degrees of freedom, a given t threshold corresponds to the same *P*-value in each, so no rescaling was required; thresholds are reported two-tailed, as in the primary analyses. The CBF map was resampled to the 3-mm grid of the functional maps, and all overlap measures were computed within the intersection of the two analysis masks. Spatial convergence was quantified using Dice and Jaccard coefficients and by a minimum-statistic conjunction, which required a reduction in both modalities within the same voxel and retained the smaller of the two absolute t values. Because the conjunction was thresholded at the voxel level without correction for multiple comparisons, the significance of the resulting overlap was assessed separately against an empirical null distribution obtained by randomly displacing the CBF map in three dimensions (5,000 iterations; minimum displacement 18 mm), a procedure that removes the spatial correspondence between the two maps while preserving the spatial autocorrelation of each. DTI regional metrics (FA, MD, AD, RD) were extracted for each of the 48 regions of the JHU atlas. Between-group differences were assessed by analysis of covariance with age, sex, and educational level as covariates, and effect sizes are reported as Hedges g. Because four diffusion metrics were tested in 48 regions across three pairwise comparisons, the resulting 576 tests were corrected using the Benjamini–Hochberg false-discovery-rate procedure; results are reported with both uncorrected *P*-values and FDR-adjusted *q*-values. These analyses were carried out in Python 3.12 using statsmodels 0.14. Regions showing significant group differences, together with the functional and perfusion indices above, were also examined in relation to BMI. Because the groups were themselves defined by BMI, correlating BMI with values extracted from clusters or tracts selected for a significant group difference is not independent evidence; such correlations are therefore reported as descriptive only. As the primary test of a dose-dependent relationship we instead used whole-sample linear regression across all 70 participants, with BMI entered as a continuous predictor and age, sex, and educational level as covariates, applied to every atlas-defined region without prior selection (48 regions × 4 diffusion metrics = 192 tests, Benjamini–Hochberg corrected). Seeds for the connectivity analysis were selected from the same dataset, so the seed-based FC analysis is likewise non-independent and is reported as exploratory. Two-sided *P* < 0.05 was considered significant. The use of different voxel-level thresholds across the functional and perfusion analyses is addressed in the Limitations.

## 3 Results

### 3.1 Participant characteristics

The three groups did not differ significantly in age, sex, educational level, or MMSE score (all *P* > 0.05) in the 70 analyzed participants, whereas BMI increased across groups by design (*P* < 0.001; Table 1). Notably, MMSE scores were at ceiling (median 30/30) in all three groups, leaving no measurable variance in global cognitive screening.

### 3.2 Resting-state function: ALFF

**FIGURE 1:**
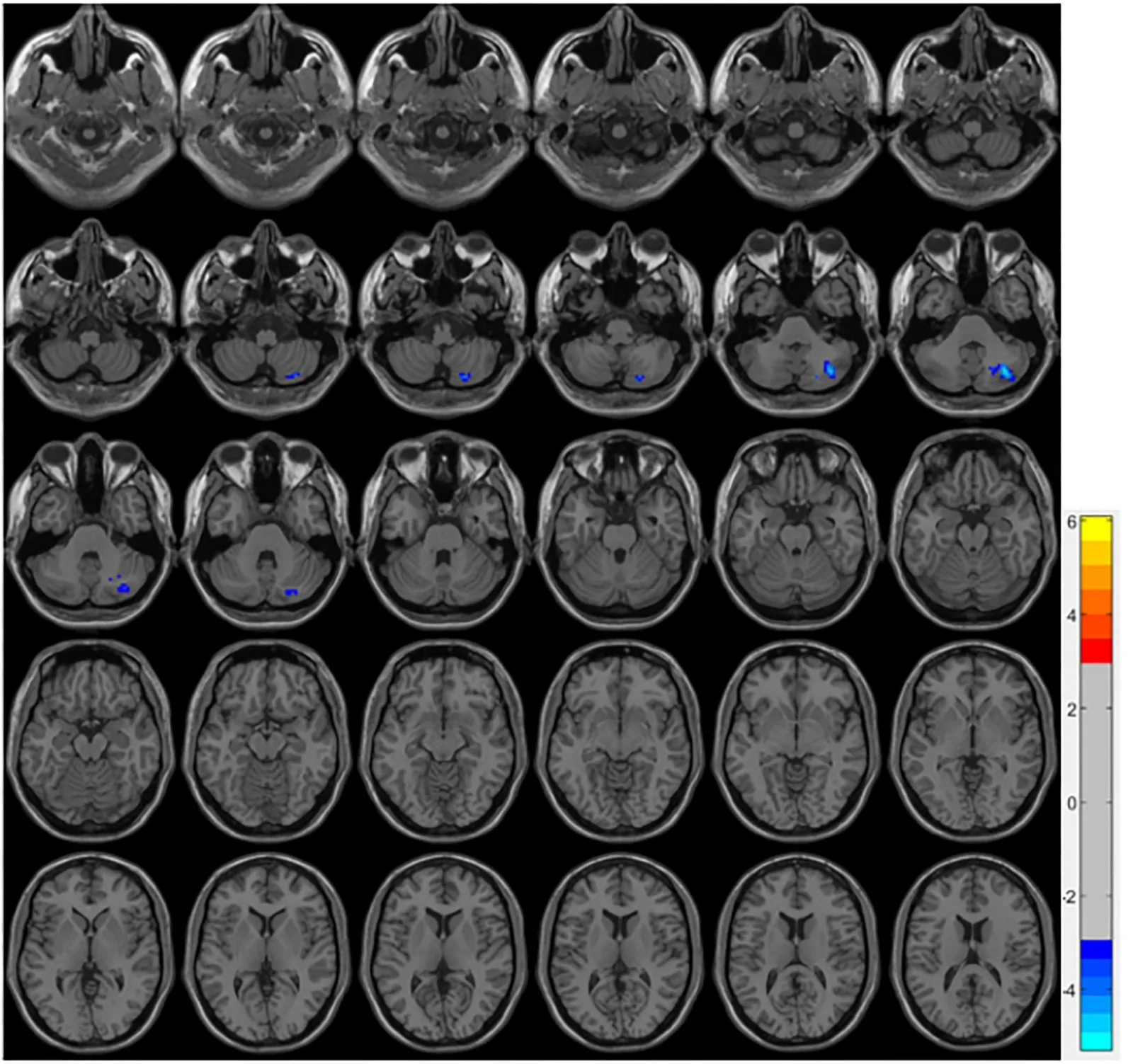
ALFF differences between the normal-weight (NW) and overweight (OW) groups. Relative to NW, the OW group showed reduced amplitude of low-frequency fluctuation (ALFF) in the right cerebellar Crus II (cool colors). Maps are overlaid on a standard T1-weighted template; the color bar denotes *t*-values (GRF-corrected, voxel-level *P* < 0.005, cluster-level *P* < 0.05).

**FIGURE 2:**
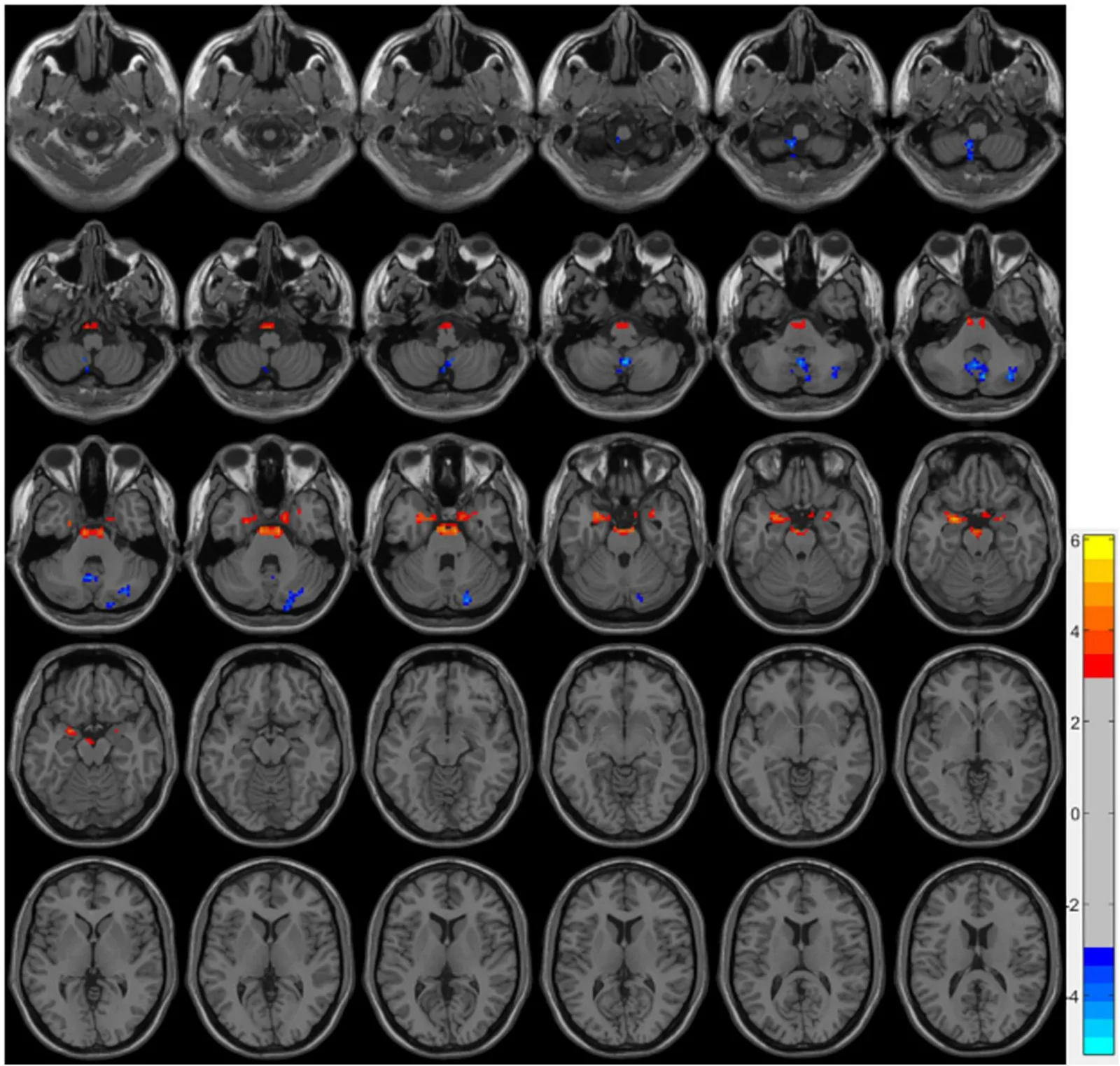
ALFF differences between the normal-weight (NW) and obese (OB) groups. Relative to NW, the OB group showed reduced ALFF in the vermis and left cerebellar lobule VIII, right cerebellar Crus II, and left superior frontal gyrus (cool colors), and increased ALFF in the bilateral parahippocampal gyri (warm colors). Display and thresholds as in Figure 1.

Compared with controls, the overweight group showed reduced ALFF in the right cerebellar Crus II. The obese group showed reduced ALFF in the vermis and left cerebellar lobule VIII, right cerebellar Crus II, and left superior frontal gyrus, and increased ALFF in the bilateral parahippocampal gyri (Table 2). ALFF in the right Crus II, the vermis and left lobule VIII, and left superior frontal gyrus correlated negatively with BMI, whereas the bilateral parahippocampal gyri correlated positively (all *P* < 0.001; Figures 1, 2 and Table 2).

**TABLE 2:** Regions showing significant ALFF differences and their correlation with BMI.

Comparison	Brain region	Cluster (voxels)	Peak MNI (x, y, z)	*T*	r (BMI)	*P*
OW < NW	Right cerebellum Crus II	86	33, −69, −39	−5.39	−0.448	< 0.001
OB < NW	Vermis and left cerebellum lobule VIII	110	0, −57, −45	−4.77	−0.659	< 0.001
OB < NW	Right cerebellum Crus II	69	33, −72, −39	−4.27	−0.448	< 0.001
OB < NW	Left superior frontal gyrus	130	−15, 39, 51	−5.31	−0.445	< 0.001
OB > NW	Left parahippocampal gyrus	82	−24, 0, −21	5.23	0.423	< 0.001
OB > NW	Right parahippocampal gyrus	55	15, 3, −30	3.79	0.438	< 0.001

GRF-corrected (voxel-level *P* < 0.005, cluster-level *P* < 0.05). Coordinates are peak MNI. The right cerebellar Crus II appears in two group comparisons; because the region was extracted once, a single correlation with BMI is reported for it rather than a separate correlation for each comparison. Correlations with BMI are descriptive only: the clusters were selected for a significant group difference and the groups were defined by BMI, so these correlations are not independent of that selection. ALFF, amplitude of low-frequency fluctuation; MNI, Montreal Neurological Institute; other abbreviations as in Table 1.

### 3.3 Resting-state function: ReHo and FC

**FIGURE 3:**
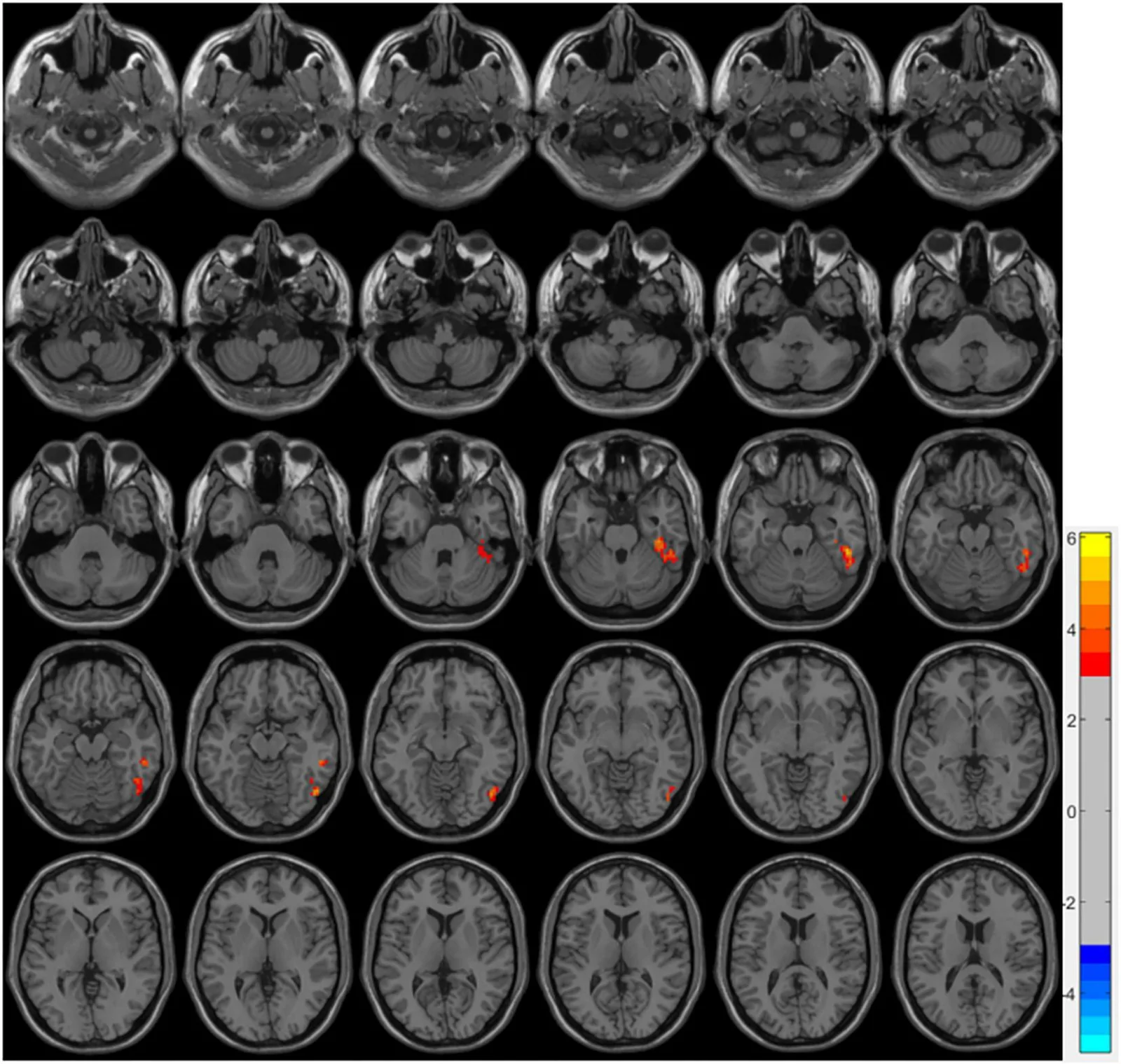
ReHo differences between the normal-weight (NW) and overweight (OW) groups. Relative to NW, the OW group showed increased regional homogeneity (ReHo) in the right inferior temporal gyrus (warm colors). Display and thresholds as in Figure 1.

**FIGURE 4:**
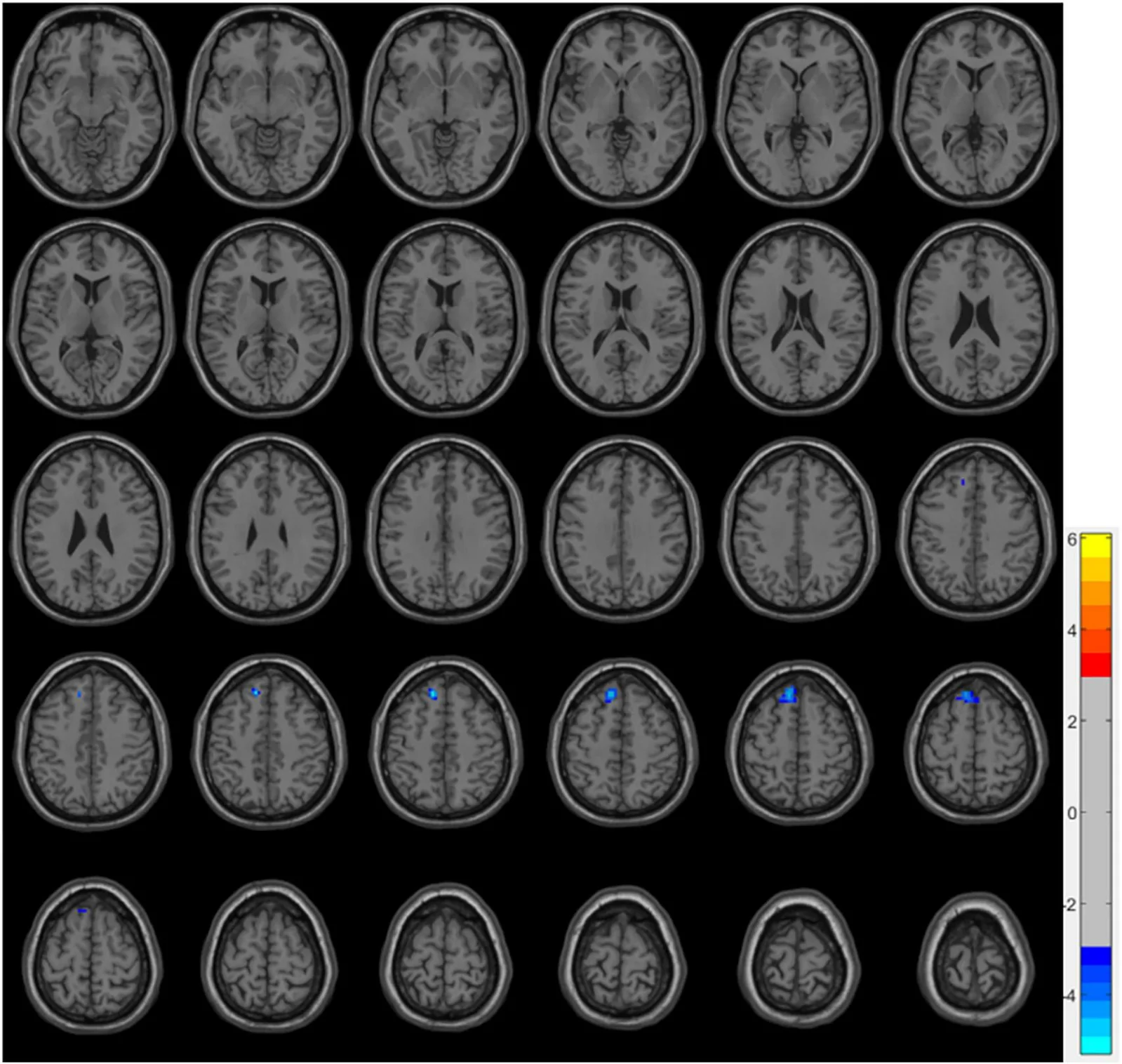
ReHo differences between the normal-weight (NW) and obese (OB) groups. Relative to NW, the OB group showed reduced ReHo in the left superior frontal gyrus (cool colors). Display and thresholds as in Figure 1.

**FIGURE 5:**
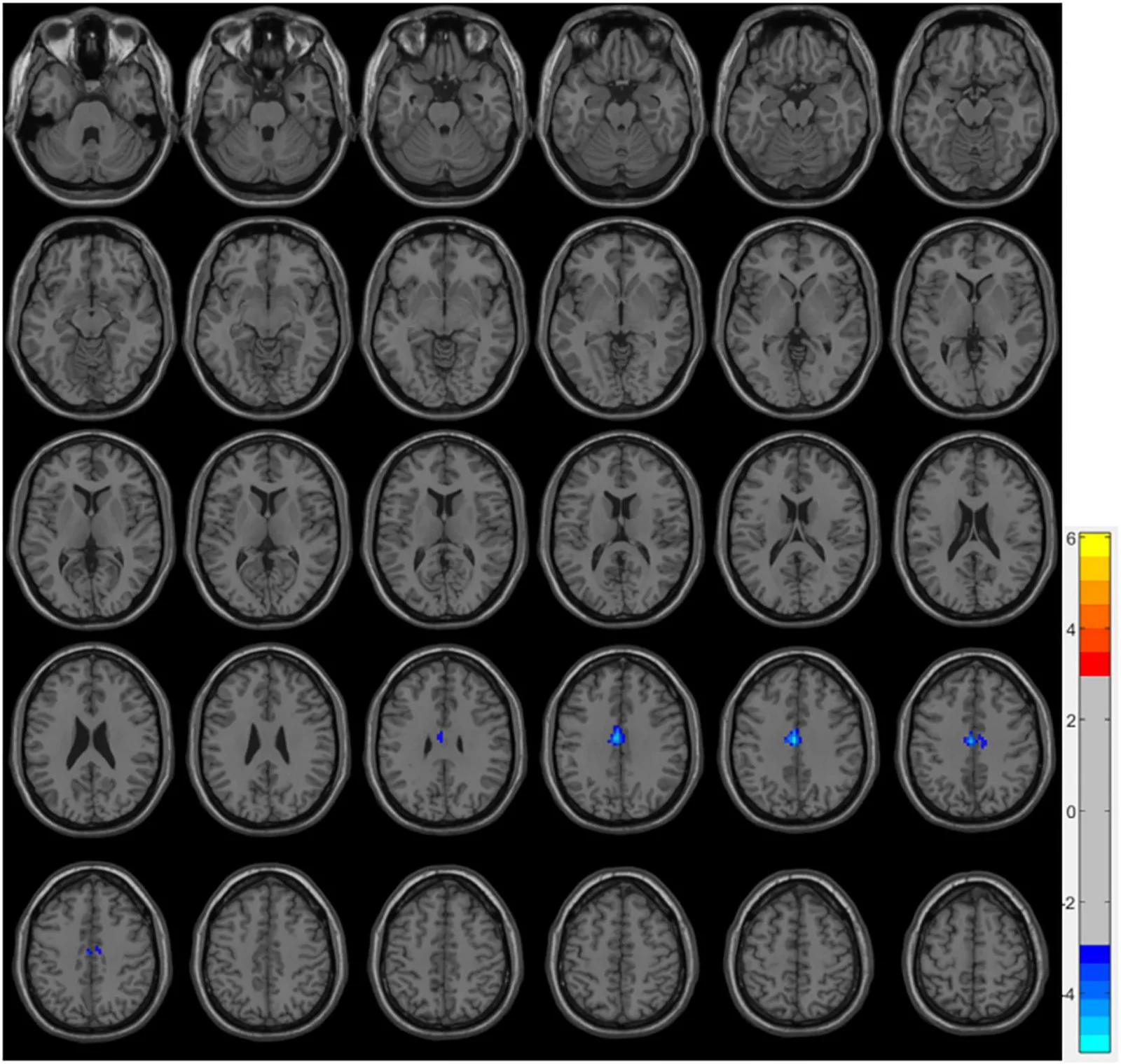
ReHo differences between the overweight (OW) and obese (OB) groups. Relative to OW, the OB group showed reduced ReHo in the left middle cingulate gyrus (cool colors). Display and thresholds as in Figure 1.

The overweight group showed higher ReHo than controls in the right inferior temporal gyrus. The obese group showed lower ReHo than controls in the left superior frontal gyrus, and lower ReHo than the overweight group in the left middle cingulate gyrus (Table 3). ReHo in the left superior frontal gyrus and left middle cingulate gyrus correlated negatively with BMI, whereas the right inferior temporal gyrus correlation was not significant (Figures 3–5 and Table 3). Seed-based FC analyses using the left superior frontal gyrus and right cerebellar Crus II as seeds revealed no significant group differences.

**TABLE 3:** Regions showing significant ReHo differences and their correlation with BMI.

Comparison	Brain region	Cluster (voxels)	Peak MNI (x, y, z)	T	r (BMI)	*P*
OW > NW	Right inferior temporal gyrus	141	51, −36, −24	4.99	0.228	0.058
OB < NW	Left superior frontal gyrus	85	−12, 36, 51	−4.97	−0.464	< 0.001
OB < OW	Left middle cingulate gyrus	85	−3, 15, 36	−6.12	−0.249	0.037

GRF-corrected (voxel-level *P* < 0.005, cluster-level *P* < 0.05). ReHo, regional homogeneity; correlations with BMI are descriptive only and are not independent of cluster selection; other abbreviations as in Tables 1, 2.

### 3.4 White-matter microstructure: DTI

At an uncorrected threshold of *P* < 0.01, the obese group differed from controls in showing reduced FA with increased MD and RD in the right inferior cerebellar peduncle, reduced FA with increased RD in the left cingulum (cingulate gyrus) and the right cingulum (hippocampal portion), and increased MD and RD in the splenium of the corpus callosum; relative to the overweight group, the obese group showed increased MD and RD in the right inferior cerebellar peduncle and higher FA in the right external capsule and the fornix. The overweight group showed higher MD than controls in the right uncinate fasciculus (Table 4). None of these regional group differences survived Benjamini-Hochberg correction across the 576 tests (smallest *q* = 0.161), and they are therefore reported as exploratory; uncorrected *P*-values, FDR-adjusted *q*-values, and effect sizes are given in Table 4. The right inferior cerebellar peduncle nevertheless stood out on three counts: it was the only tract altered in three separate metrics (FA, MD, and RD) in both the obese-versus-normal-weight and obese-versus-overweight comparisons at an uncorrected *P* < 0.05 (the FA difference between the obese and overweight groups, *P* = 0.028, falls between the two thresholds and is therefore not listed in Table 4); the effect sizes were large and graded with adiposity (Hedges g = −1.12 for FA, 0.99 for MD, and 1.10 for RD in the obese-versus-normal-weight comparison, and −0.69, 0.81, and 0.84 respectively in the obese-versus-overweight comparison); and the change in RD substantially exceeded that in AD (*g* = 0.59, *P* = 0.083). In the whole-sample regression, in which BMI was entered as a continuous predictor and every atlas region was tested without prior selection, the right inferior cerebellar peduncle provided the only associations to survive correction: BMI predicted FA (standardized beta = −0.46, *q* = 0.009), RD (beta = 0.44, *q* = 0.009), and MD (beta = 0.41, *q* = 0.014) in this tract. Because this analysis does not use the BMI-defined grouping, it is not subject to the circularity affecting the within-tract correlations, which are therefore no longer reported.

**TABLE 4:** Associations between white-matter diffusion metrics and adiposity.

Panel A. Primary analysis: whole-sample regression on BMI				
Metric	Tract (JHU)	Standardized β	*P*	*q*
RD	Right inferior cerebellar peduncle	+0.442	0.000067	0.009
FA	Right inferior cerebellar peduncle	−0.461	0.000096	0.009
MD	Right inferior cerebellar peduncle	+0.405	0.00022	0.014
**Panel B. Exploratory between-group comparisons**				
**Metric**	**Tract (JHU)**	**Comparison**	**Hedges g**	***P* (*q*)**
RD	Right cingulum (hippocampal)	OB > NW	+0.95	0.0007 (0.161)
RD	Right inferior cerebellar peduncle	OB > NW	+1.10	0.0007 (0.161)
FA	Right inferior cerebellar peduncle	OB < NW	−1.12	0.0010 (0.161)
RD	Left cingulum (cingulate gyrus)	OB > NW	+1.03	0.0012 (0.161)
RD	Splenium of corpus callosum	OB > NW	+0.90	0.0014 (0.161)
MD	Right inferior cerebellar peduncle	OB > NW	+0.99	0.0025 (0.211)
FA	Left cingulum (cingulate gyrus)	OB < NW	−0.96	0.0028 (0.211)
FA	Right cingulum (hippocampal)	OB < NW	−0.85	0.0029 (0.211)
MD	Right uncinate fasciculus	OW > NW	+0.67	0.0043 (0.244)
FA	Right external capsule	OB > OW	+0.64	0.0045 (0.244)
MD	Splenium of corpus callosum	OB > NW	+0.83	0.0050 (0.244)
RD	Right inferior cerebellar peduncle	OB > OW	+0.84	0.0051 (0.244)
MD	Right inferior cerebellar peduncle	OB > OW	+0.81	0.0073 (0.325)
FA	Fornix	OB > OW	+0.64	0.0080 (0.327)

Panel A: whole-sample linear regression across all 70 participants with BMI entered as a continuous predictor and age, sex and educational level as covariates, applied to all 48 atlas regions and four diffusion metrics (192 tests, Benjamini–Hochberg corrected); only associations with q < 0.05 are shown, and full results for all 192 tests are given in Supplementary Table 2. Panel B: analysis of covariance with the same covariates across the three pairwise group comparisons (576 tests); no comparison survived FDR correction (smallest *q* = 0.161), and regions with uncorrected *P* < 0.01 are listed, with full results for all 576 tests given in Supplementary Table 1. Panel A reports standardized regression coefficients; because BMI was not used to define groups and all atlas regions were tested without prior selection, these estimates are not subject to selection bias. Panel B reports exploratory between-group comparisons with uncorrected *P*-values, FDR-adjusted *q*-values in parentheses, and Hedges g as the effect size; no comparison in Panel B survived correction, and these results should not be interpreted as significant. Correlations between BMI and values extracted from tracts selected for a significant group difference are not independent of that selection and are therefore no longer reported. FA, fractional anisotropy; MD, mean diffusivity; AD, axial diffusivity; RD, radial diffusivity; JHU, Johns Hopkins University; other abbreviations as in Table 1.

### 3.5 Cerebral perfusion: CBF

**FIGURE 6:**
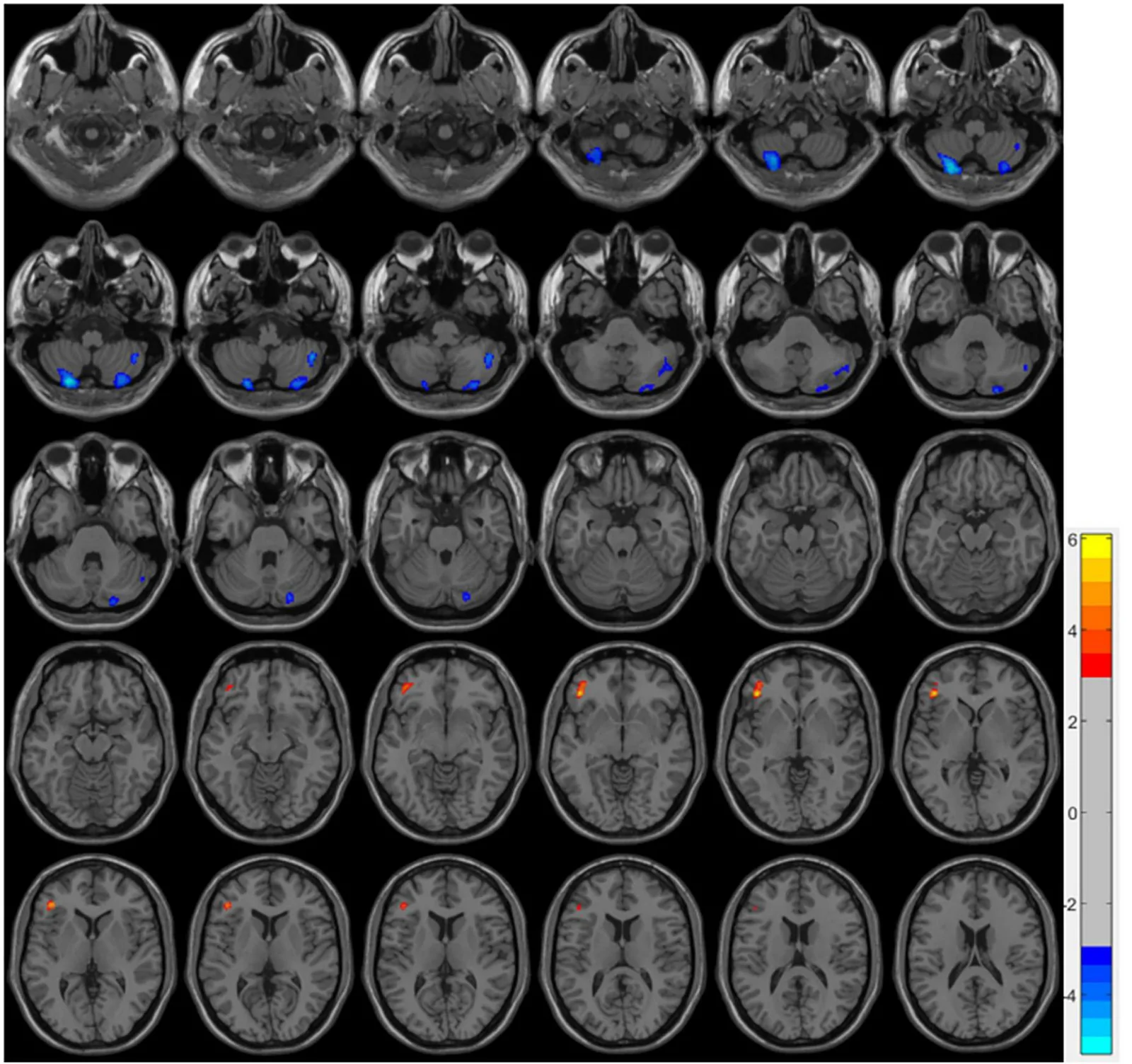
CBF differences between the normal-weight (NW) and obese (OB) groups. Relative to NW, the OB group showed reduced cerebral blood flow (CBF) in the left cerebellar lobule VIII and right cerebellar Crus I/II (cool colors), and increased CBF in the triangular part of the left inferior frontal gyrus and the right postcentral gyrus (warm colors). Maps are overlaid on a standard T1-weighted template; the color bar denotes *t*-values (GRF-corrected, voxel-level *P* < 0.001, cluster-level *P* < 0.05).

Compared with controls, the obese group showed reduced CBF in the left cerebellar lobule VIII, right cerebellar Crus I, and right cerebellar Crus II, and increased CBF in the triangular part of the left inferior frontal gyrus and the right postcentral gyrus (Table 5). Cerebellar CBF correlated negatively with BMI, whereas the inferior frontal and postcentral values correlated positively (all *P* < 0.001; Figure 6 and Table 5).

**TABLE 5:** Regions showing significant CBF differences and their correlation with BMI.

Comparison	Brain region	Cluster (voxels)	Peak MNI (x, y, z)	*T*	r (BMI)	*P*
OB < NW	Left cerebellum lobule VIII	437	−24, −78, −50	−5.13	−0.432	< 0.001
OB < NW	Right cerebellum Crus II	388	30, −80, −48	−3.98	−0.416	< 0.001
OB < NW	Right cerebellum Crus I	192	44, −52, −46	−4.14	−0.442	< 0.001
OB > NW	Left inferior frontal gyrus (triangular)	229	−42, 36, 0	5.16	0.517	< 0.001
OB > NW	Right postcentral gyrus	198	30, −36, 70	4.43	0.467	< 0.001

GRF-corrected (voxel-level *P* < 0.001, cluster-level *P* < 0.05). Correlations with BMI are descriptive only and are not independent of cluster selection; CBF values were scaled to the whole-brain mean gray-matter value and therefore represent relative perfusion. CBF, cerebral blood flow; other abbreviations as in Tables 1, 2.

### 3.6 Convergent multimodal abnormalities

Several regions showed abnormalities across more than one modality. The cerebellar posterior lobe showed concordant ALFF and CBF reductions in the obese group. Overlap between the two modalities exceeded chance expectation at every threshold examined, and the excess grew as the threshold became more stringent (4.6-fold at *P* < 0.05, 14.2-fold at *P* < 0.01, 22.0-fold at *P* < 0.005, and 43.1-fold at *P* < 0.001). A random-displacement permutation test confirmed that the observed overlap exceeded the null distribution at every threshold examined (empirical *P* = 0.0002 at *P* < 0.05 and *P* < 0.005, and *P* = 0.0008 at *P* < 0.001). A minimum-statistic conjunction, thresholded at an uncorrected voxel-level *P* < 0.001 in both modalities, retained 13 voxels in three clusters with its peak in the left cerebellar lobule VIII (minimum *t* = 4.48 at −24, −75, −51); at a voxel-level *P* < 0.05 the two largest conjunction clusters were centered on the right cerebellar Crus II extending into lobule VIII (319 voxels; minimum *t* = 3.68 at 30, −75, −51) and on the left cerebellar lobule VIII (165 voxels). For the right Crus II, both modalities are represented by clusters reported independently in Tables 2, 5. For the left cerebellar lobule VIII, the CBF cluster is reported in Table 5, whereas the corresponding ALFF cluster (peak t = −5.19 at −27, −75, −48) did not survive cluster-level correction in the primary analysis and is therefore not listed in Table 2, notwithstanding that it contains the strongest cerebellar ALFF peak in the map. At the vermal ALFF peak (0, −57, −45), by contrast, the corresponding CBF effect was negligible (*t* = −0.97), so the apparent overlap at that site reflects shared membership of the same atlas-defined (AAL) region rather than co-located peaks. Voxel counts above refer to reductions only, so they are not expected to approach the nominal two-tailed rate. The remaining multimodal correspondences require a different and weaker description. The left superior frontal gyrus showed reduced ALFF and reduced ReHo with peaks 4.2 mm apart, the closest spatial correspondence in the study; because ALFF and ReHo are computed from the same resting-state data, however, their agreement is not independent evidence and is not comparable to the ALFF-CBF convergence. The cingulum bundle showed changes in more than one diffusion metric, but FA, MD, AD, and RD are derived from the same tensor and likewise do not constitute independent modalities. Evidence for each convergent region, graded as peak-level, atlas-region-level, or non-independent, is summarized in Supplementary Table 3. The right inferior cerebellar peduncle showed a coherent white-matter pattern of reduced FA together with increased MD and RD, with graded MD changes across groups.

## 4 Discussion

Using a multimodal MRI protocol in a demographically matched cohort spanning the normal-weight–overweight–obese spectrum, we found that overweight and obesity are associated with functional, white-matter microstructural, and perfusion abnormalities that, importantly, converge within the same regions—most consistently the cerebellar posterior lobe and the right inferior cerebellar peduncle, with weaker and non-independent correspondences in the prefrontal cortex and cingulum. In whole-sample regression, adiposity was related continuously to diffusion metrics in the right inferior cerebellar peduncle; the remaining associations with BMI did not survive correction. The principal contribution of this study is the demonstration of spatially convergent, cross-modal abnormalities within a single cohort, which strengthens the inference that these are coherent, obesity-related changes rather than modality-specific or incidental findings.

Cerebellar involvement was a prominent and recurring feature. The posterior lobe (the left lobule VIII and the right Crus II) showed concurrent reductions in spontaneous activity (ALFF) and perfusion (CBF) (30), and adjacent white-matter pathways—the inferior and middle cerebellar peduncles—showed reduced FA with increased MD and RD in the inferior peduncle, and increased MD and RD without a significant FA change in the middle peduncle (the middle-peduncle differences reached only an uncorrected *P* < 0.05 and fall below the *P* < 0.01 cut-off used for Table 4). Beyond its classical motor role, the cerebellum participates in cognitive and affective processing (31), and structural and volumetric differences in obesity have been reported across cortical and subcortical regions including the cerebellum (32, 33), some of which correlate with BMI. The right inferior cerebellar peduncle was particularly notable, showing reduced FA together with increased MD and RD and graded MD changes across groups—a combination consistent with concurrent disruption of fiber integrity and myelin, and suggesting that this tract may be an early and sensitive site of obesity-related white-matter change. This tract was also the only site at which the association with BMI survived correction for multiple comparisons: in whole-sample regression across all 70 participants, with age, sex, and educational level as covariates and no prior selection of regions, BMI predicted FA (standardized beta = −0.46), RD (beta = 0.44), and MD (beta = 0.41) in the right inferior cerebellar peduncle (all *q* < 0.015, Benjamini-Hochberg corrected across 192 tests). Because BMI was treated as a continuous variable and every atlas-defined region was tested, this analysis does not depend on the BMI-defined grouping and is therefore not subject to the circularity that affects the within-cluster correlations reported above.

Changes in more than one index were also seen in the prefrontal cortex and cingulum, although these do not constitute cross-modal convergence in the sense established above: the left superior frontal gyrus showed reductions in both ALFF and ReHo, which derive from the same resting-state data, and the cingulum showed changes in several diffusion metrics derived from the same tensor together with altered middle cingulate ReHo, at uncorrected thresholds only. The prefrontal cortex and cingulate are central to executive and inhibitory control and to emotion regulation, and their involvement features prominently in accounts of obesity-related brain change (8, 9, 34). Reduced FA in tracts such as the corpus callosum, superior longitudinal fasciculus, and cingulum—all of which reached only an uncorrected *P* < 0.05 here and are therefore exploratory—is consistent with previous diffusion studies in obesity (16). The increases we observed—higher ALFF in the parahippocampal gyri, higher ReHo in the inferior temporal gyrus, and higher CBF in the inferior frontal and postcentral gyri—may reflect compensatory or adaptive responses, in line with reports that some regions show increased rather than decreased activity in obesity (10), although this interpretation is tentative and requires confirmation.

The four DTI metrics together provide a more complete characterization of white-matter change than FA and MD alone. Elevations in RD that exceeded the corresponding changes in AD were the predominant pattern across the affected tracts, which is compatible with a myelin-related process. This inference should not be pressed too far, however: the tensor model averages over all fiber populations within a voxel, so in regions containing crossing or fanning fibers the relationship between RD and myelin is not specific. The AD findings were mixed (both increases and decreases), which may reflect regional heterogeneity, partial-volume effects, or crossing fibers. Advanced microstructural models such as neurite orientation dispersion and density imaging would be required to separate axonal from myelin contributions, and we therefore regard the present diffusion findings as consistent with, rather than demonstrating, altered myelination. A further consideration concerns the choice of white-matter parcellation. We used the JHU ICBM-DTI-81 atlas, which delineates tracts from diffusion anatomy alone. A recently published multimodal white-matter atlas (MWMA) instead parcellates white matter by combining local anatomical architecture with global functional dynamics: its boundaries are generated by two-level spectral clustering of a similarity network built from six imaging features—the amplitude of low-frequency fluctuation, dynamic ALFF, degree centrality and regional homogeneity from resting-state fMRI, together with fractional anisotropy and mean diffusivity from diffusion imaging—and were validated against imaging-feature and neurotransmitter similarity transitions (35). Because its parcels are defined partly by functional signal, the MWMA is in principle better matched to a study such as ours in which functional and microstructural findings are compared, and it has been reported to describe the white matter’s cerebellum-to-association gradient more faithfully than a tract-based atlas—a distinction that bears directly on the cerebellar peduncular findings reported here. We have retained the JHU parcellation for the present analysis because it was specified in advance, because the MWMA was derived from 7-T Human Connectome Project data whereas ours are 3-T clinical acquisitions with 2-mm isotropic diffusion resolution, and because changing the parcellation at revision would alter which tracts are reported. Re-examining these data under the MWMA is nevertheless a clear direction for future work. Although the hypothalamus has been implicated in the neurobiology of obesity (36), our atlas-based tract analysis did not specifically target this region. The tracts implicated here—corpus callosum, superior longitudinal fasciculus, cingulum, and cerebellar peduncles—connect regions belonging to large-scale executive, default-mode, and sensorimotor networks, consistent with a distributed rather than focal pattern of involvement.

A graded relationship with adiposity was suggested but is more limited than our original analysis implied. In the right inferior cerebellar peduncle the association with BMI was continuous across the whole sample and survived correction for multiple comparisons, and the group contrasts in that tract were ordered obese > overweight > normal-weight. The changes detectable in the overweight group elsewhere, however, did not survive correction and are exploratory. Any dose-response interpretation therefore rests on the cerebellar peduncle alone, and the cross-sectional design precludes causal inference.

An important consideration concerns the functional significance of these findings. The affected regions and tracts are known to subserve executive, affective, and sensorimotor functions, and obesity-related changes in such regions have been discussed in relation to cognition (17); it is therefore tempting to interpret their disruption as evidence of cognitive decline. However, cognition in this study was screened only with the MMSE, which showed a ceiling effect (median 30/30 in all groups) and is, in any case, a dementia-screening instrument insensitive to the subtle changes expected in relatively young and cognitively healthy adults; no sensitive neuropsychological battery was administered. We therefore deliberately refrain from concluding that the observed imaging abnormalities are accompanied by measurable cognitive impairment. Any cognitive interpretation is inferential, based on the established functional roles of the affected regions, and must be tested directly in future work.

From a clinical perspective, multimodal indices—particularly those at convergent sites such as the right inferior cerebellar peduncle and the cerebellar posterior lobe—may serve as candidate imaging markers of early, subclinical brain involvement in overweight and obesity, and could in principle help monitor brain health or treatment response, including after weight-loss interventions (37, 38). This potential remains hypothesis-generating and requires validation in larger, longitudinal, and interventional studies.

### 4.1 Limitations

This study has several limitations. First, the sample was modest, and after division into three groups the per-group sizes were small, which limits statistical power—particularly for the voxel-wise functional and perfusion analyses—and means that some findings may not survive more stringent correction; the seed-based FC analysis, which was null, may have been underpowered or affected by residual physiological noise. Inference for the voxel-wise functional and perfusion maps remains parametric: cluster-level correction was performed with Gaussian random field theory, and a full voxel-wise permutation analysis of the individual maps was not undertaken. Non-parametric inference was applied only to the test of spatial convergence. A further consequence of the ASL processing is that CBF maps were scaled to the whole-brain mean gray-matter value, so the perfusion findings describe relative rather than absolute cerebral blood flow, and a uniform global difference between groups would not be detected by this analysis. Second, different voxel-level thresholds were used for the functional (*P* < 0.005) and perfusion (*P* < 0.001) analyses, reflecting standard pipelines. To ensure that this did not generate the convergence findings, the overlap between modalities was re-examined at thresholds common to both and tested formally by conjunction and permutation; the convergence persisted and became proportionally stronger at more stringent thresholds. Residual differences in sensitivity between the two pipelines nevertheless remain. Third, obesity was defined solely by BMI, and no biochemical or metabolic data (e.g., glucose, insulin, lipids, HOMA-IR, waist circumference, or visceral adiposity) were collected; we were therefore unable to account for metabolic phenotype or to distinguish metabolically healthy from unhealthy obesity. All participants were scanned in the postprandial state and none had diagnosed diabetes, but blood pressure, blood glucose concentration, medication use, and menstrual-cycle phase in female participants were not recorded. Each of these can influence cerebral blood flow and the BOLD signal, and they are not excluded by our criteria; their potential contribution to the perfusion and functional findings therefore cannot be assessed. In addition, none of the between-group differences in the diffusion metrics survived false-discovery-rate correction across the 576 regional tests, so the regional group comparisons should be regarded as exploratory; only the whole-sample associations with BMI in the right inferior cerebellar peduncle survived correction. Finally, the seeds for the connectivity analysis were defined from the same dataset, so that analysis is not independent of the regional findings that motivated it. Fourth, cognition was assessed only by the MMSE, which showed a ceiling effect and is insensitive to subtle change, so the cognitive relevance of the imaging findings could not be established. Fifth, the cross-sectional design precludes causal inference and does not capture changes after weight loss. Finally, other lifestyle factors such as diet and physical activity were not quantified. Future studies should enlarge and prospectively follow the sample, incorporate sensitive neuropsychological testing and metabolic profiling, and add complementary modalities (e.g., MR spectroscopy) to clarify the mechanisms and functional consequences of obesity-related brain changes.

## 5 Conclusion

In a demographically matched cohort, overweight and obesity were associated with spatially convergent functional, white-matter microstructural, and perfusion abnormalities that preferentially involved cerebellar and cortico-limbic regions. The only association with adiposity to survive correction for multiple comparisons was in the right inferior cerebellar peduncle, where fractional anisotropy, mean diffusivity and radial diffusivity varied continuously with BMI across the whole sample. The cerebellar posterior lobe (concordant ALFF and CBF reductions) and the right inferior cerebellar peduncle (reduced FA with increased MD and RD) emerged as the most consistent convergent sites and represent promising candidate imaging markers of early brain involvement. Because cognition was not assessed with a sensitive battery, the consequences of these changes for cognition remain to be determined and should be a focus of future longitudinal, metabolically characterized studies.

## Data Availability

The original contributions presented in the study are included in the article/Supplementary material, further inquiries can be directed to the corresponding authors.
