# Supplementary material for: Convergent functional, white-matter microstructural, and cerebral perfusion abnormalities in overweight and obese adults: a multimodal MRI study
Source: Front Nutr. 2026 Sep 3;13:1917977. doi: 10.3389/fnut.2026.1917977 (PMC13581818; doi:10.3389/fnut.2026.1917977)
Supplement: Supplementary file 1 [file Table_1.docx]

**Supplementary Material**

# **Supplementary Table S1. Complete results of the regional diffusion analysis**

Analysis of covariance with age, sex, and educational level as covariates, for each of the 48 regions of the JHU ICBM-DTI-81 atlas and each of the four diffusion metrics, across the three pairwise group comparisons (n = 70; 27 normal-weight, 21 overweight, 22 obese). All 576 tests are listed. P values are uncorrected; q values are Benjamini–Hochberg adjusted across the full family of 576 tests. No comparison survived correction (smallest q = 0.161). Hedges g is the standardised effect size, with a positive sign indicating a higher value in the second-named group.

| **Metric** | **Tract (JHU)** | **Comparison** | **Hedges g** | **P** | **q** |
| --- | --- | --- | --- | --- | --- |
| FA | Middle cerebellar peduncle | NW vs OW | -0.27 | 0.1093 | 0.702 |
| FA | Middle cerebellar peduncle | NW vs OB | -0.49 | 0.1318 | 0.717 |
| FA | Middle cerebellar peduncle | OW vs OB | -0.25 | 0.6389 | 0.938 |
| FA | Pontine crossing tract | NW vs OW | -0.43 | 0.1600 | 0.762 |
| FA | Pontine crossing tract | NW vs OB | -0.36 | 0.2829 | 0.813 |
| FA | Pontine crossing tract | OW vs OB | +0.03 | 0.5729 | 0.914 |
| FA | Genu of corpus callosum | NW vs OW | +0.01 | 0.7508 | 0.938 |
| FA | Genu of corpus callosum | NW vs OB | +0.40 | 0.0870 | 0.659 |
| FA | Genu of corpus callosum | OW vs OB | +0.41 | 0.2569 | 0.813 |
| FA | Body of corpus callosum | NW vs OW | -0.46 | 0.1882 | 0.793 |
| FA | Body of corpus callosum | NW vs OB | -0.74 | 0.0104 | 0.376 |
| FA | Body of corpus callosum | OW vs OB | -0.16 | 0.4965 | 0.906 |
| FA | Splenium of corpus callosum | NW vs OW | -0.00 | 0.7619 | 0.938 |
| FA | Splenium of corpus callosum | NW vs OB | -0.53 | 0.0588 | 0.658 |
| FA | Splenium of corpus callosum | OW vs OB | -0.45 | 0.0721 | 0.659 |
| FA | Fornix | NW vs OW | -0.61 | 0.0457 | 0.643 |
| FA | Fornix | NW vs OB | +0.06 | 0.6717 | 0.938 |
| FA | Fornix | OW vs OB | +0.64 | 0.0080 | 0.327 |
| FA | Corticospinal tract R | NW vs OW | +0.37 | 0.1742 | 0.767 |
| FA | Corticospinal tract R | NW vs OB | -0.02 | 0.8457 | 0.952 |
| FA | Corticospinal tract R | OW vs OB | -0.34 | 0.4703 | 0.906 |
| FA | Corticospinal tract L | NW vs OW | +0.53 | 0.0542 | 0.648 |
| FA | Corticospinal tract L | NW vs OB | +0.48 | 0.0661 | 0.659 |
| FA | Corticospinal tract L | OW vs OB | -0.02 | 0.5080 | 0.906 |
| FA | Medial lemniscus R | NW vs OW | +0.06 | 0.7896 | 0.938 |
| FA | Medial lemniscus R | NW vs OB | +0.07 | 0.7517 | 0.938 |
| FA | Medial lemniscus R | OW vs OB | +0.01 | 0.8166 | 0.949 |
| FA | Medial lemniscus L | NW vs OW | +0.24 | 0.4941 | 0.906 |
| FA | Medial lemniscus L | NW vs OB | +0.23 | 0.3247 | 0.846 |
| FA | Medial lemniscus L | OW vs OB | -0.02 | 0.7767 | 0.938 |
| FA | Inferior cerebellar peduncle R | NW vs OW | -0.38 | 0.3235 | 0.846 |
| FA | Inferior cerebellar peduncle R | NW vs OB | -1.12 | 0.0010 | 0.161 |
| FA | Inferior cerebellar peduncle R | OW vs OB | -0.69 | 0.0283 | 0.598 |
| FA | Inferior cerebellar peduncle L | NW vs OW | -0.02 | 0.9100 | 0.966 |
| FA | Inferior cerebellar peduncle L | NW vs OB | -0.62 | 0.0452 | 0.643 |
| FA | Inferior cerebellar peduncle L | OW vs OB | -0.67 | 0.0752 | 0.659 |
| FA | Superior cerebellar peduncle R | NW vs OW | -0.13 | 0.2405 | 0.813 |
| FA | Superior cerebellar peduncle R | NW vs OB | -0.41 | 0.2014 | 0.794 |
| FA | Superior cerebellar peduncle R | OW vs OB | -0.25 | 0.8405 | 0.952 |
| FA | Superior cerebellar peduncle L | NW vs OW | +0.06 | 0.6693 | 0.938 |
| FA | Superior cerebellar peduncle L | NW vs OB | -0.31 | 0.3132 | 0.836 |
| FA | Superior cerebellar peduncle L | OW vs OB | -0.32 | 0.8571 | 0.952 |
| FA | Cerebral peduncle R | NW vs OW | +0.32 | 0.2507 | 0.813 |
| FA | Cerebral peduncle R | NW vs OB | +0.47 | 0.1320 | 0.717 |
| FA | Cerebral peduncle R | OW vs OB | +0.13 | 0.9132 | 0.966 |
| FA | Cerebral peduncle L | NW vs OW | +0.03 | 0.8102 | 0.948 |
| FA | Cerebral peduncle L | NW vs OB | +0.61 | 0.0444 | 0.643 |
| FA | Cerebral peduncle L | OW vs OB | +0.62 | 0.0828 | 0.659 |
| FA | Anterior limb of internal capsule R | NW vs OW | -0.50 | 0.1141 | 0.702 |
| FA | Anterior limb of internal capsule R | NW vs OB | +0.47 | 0.2194 | 0.811 |
| FA | Anterior limb of internal capsule R | OW vs OB | +0.91 | 0.0175 | 0.439 |
| FA | Anterior limb of internal capsule L | NW vs OW | -0.35 | 0.2470 | 0.813 |
| FA | Anterior limb of internal capsule L | NW vs OB | +0.36 | 0.3375 | 0.849 |
| FA | Anterior limb of internal capsule L | OW vs OB | +0.73 | 0.0524 | 0.643 |
| FA | Posterior limb of internal capsule R | NW vs OW | -0.00 | 0.7815 | 0.938 |
| FA | Posterior limb of internal capsule R | NW vs OB | +0.39 | 0.2352 | 0.813 |
| FA | Posterior limb of internal capsule R | OW vs OB | +0.35 | 0.4783 | 0.906 |
| FA | Posterior limb of internal capsule L | NW vs OW | -0.01 | 0.9651 | 0.990 |
| FA | Posterior limb of internal capsule L | NW vs OB | +0.15 | 0.5392 | 0.906 |
| FA | Posterior limb of internal capsule L | OW vs OB | +0.15 | 0.7288 | 0.938 |
| FA | Retrolenticular internal capsule R | NW vs OW | -0.47 | 0.1758 | 0.767 |
| FA | Retrolenticular internal capsule R | NW vs OB | -0.08 | 0.7444 | 0.938 |
| FA | Retrolenticular internal capsule R | OW vs OB | +0.43 | 0.2964 | 0.832 |
| FA | Retrolenticular internal capsule L | NW vs OW | -0.19 | 0.4280 | 0.892 |
| FA | Retrolenticular internal capsule L | NW vs OB | +0.17 | 0.4248 | 0.892 |
| FA | Retrolenticular internal capsule L | OW vs OB | +0.37 | 0.3594 | 0.855 |
| FA | Anterior corona radiata R | NW vs OW | -0.15 | 0.8247 | 0.950 |
| FA | Anterior corona radiata R | NW vs OB | -0.44 | 0.1668 | 0.767 |
| FA | Anterior corona radiata R | OW vs OB | -0.26 | 0.2892 | 0.825 |
| FA | Anterior corona radiata L | NW vs OW | -0.35 | 0.4287 | 0.892 |
| FA | Anterior corona radiata L | NW vs OB | -0.33 | 0.3541 | 0.855 |
| FA | Anterior corona radiata L | OW vs OB | +0.05 | 0.6632 | 0.938 |
| FA | Superior corona radiata R | NW vs OW | -0.59 | 0.0505 | 0.643 |
| FA | Superior corona radiata R | NW vs OB | -0.27 | 0.2721 | 0.813 |
| FA | Superior corona radiata R | OW vs OB | +0.35 | 0.3343 | 0.849 |
| FA | Superior corona radiata L | NW vs OW | -0.55 | 0.0912 | 0.664 |
| FA | Superior corona radiata L | NW vs OB | -0.26 | 0.2455 | 0.813 |
| FA | Superior corona radiata L | OW vs OB | +0.28 | 0.7779 | 0.938 |
| FA | Posterior corona radiata R | NW vs OW | -0.41 | 0.1501 | 0.753 |
| FA | Posterior corona radiata R | NW vs OB | -0.24 | 0.1886 | 0.793 |
| FA | Posterior corona radiata R | OW vs OB | +0.12 | 0.9968 | 0.997 |
| FA | Posterior corona radiata L | NW vs OW | -0.29 | 0.3296 | 0.849 |
| FA | Posterior corona radiata L | NW vs OB | -0.31 | 0.1292 | 0.716 |
| FA | Posterior corona radiata L | OW vs OB | -0.04 | 0.8241 | 0.950 |
| FA | Posterior thalamic radiation R | NW vs OW | -0.27 | 0.4632 | 0.906 |
| FA | Posterior thalamic radiation R | NW vs OB | -0.37 | 0.1544 | 0.753 |
| FA | Posterior thalamic radiation R | OW vs OB | -0.14 | 0.6378 | 0.938 |
| FA | Posterior thalamic radiation L | NW vs OW | -0.30 | 0.5476 | 0.906 |
| FA | Posterior thalamic radiation L | NW vs OB | -0.27 | 0.2982 | 0.832 |
| FA | Posterior thalamic radiation L | OW vs OB | +0.01 | 0.7792 | 0.938 |
| FA | Sagittal stratum R | NW vs OW | -0.80 | 0.0154 | 0.432 |
| FA | Sagittal stratum R | NW vs OB | -0.34 | 0.2371 | 0.813 |
| FA | Sagittal stratum R | OW vs OB | +0.38 | 0.3143 | 0.836 |
| FA | Sagittal stratum L | NW vs OW | -0.71 | 0.0312 | 0.598 |
| FA | Sagittal stratum L | NW vs OB | -0.13 | 0.5466 | 0.906 |
| FA | Sagittal stratum L | OW vs OB | +0.50 | 0.1928 | 0.793 |
| FA | External capsule R | NW vs OW | -0.06 | 0.6078 | 0.927 |
| FA | External capsule R | NW vs OB | +0.49 | 0.0765 | 0.659 |
| FA | External capsule R | OW vs OB | +0.64 | 0.0045 | 0.244 |
| FA | External capsule L | NW vs OW | +0.07 | 0.9663 | 0.990 |
| FA | External capsule L | NW vs OB | +0.46 | 0.1158 | 0.702 |
| FA | External capsule L | OW vs OB | +0.41 | 0.0671 | 0.659 |
| FA | Cingulum (cingulate gyrus) R | NW vs OW | -0.31 | 0.2517 | 0.813 |
| FA | Cingulum (cingulate gyrus) R | NW vs OB | -0.67 | 0.0337 | 0.598 |
| FA | Cingulum (cingulate gyrus) R | OW vs OB | -0.31 | 0.6485 | 0.938 |
| FA | Cingulum (cingulate gyrus) L | NW vs OW | -0.44 | 0.1948 | 0.793 |
| FA | Cingulum (cingulate gyrus) L | NW vs OB | -0.96 | 0.0028 | 0.211 |
| FA | Cingulum (cingulate gyrus) L | OW vs OB | -0.33 | 0.4397 | 0.894 |
| FA | Cingulum (hippocampal) R | NW vs OW | -0.34 | 0.3668 | 0.870 |
| FA | Cingulum (hippocampal) R | NW vs OB | -0.85 | 0.0029 | 0.211 |
| FA | Cingulum (hippocampal) R | OW vs OB | -0.34 | 0.0769 | 0.659 |
| FA | Cingulum (hippocampal) L | NW vs OW | -0.36 | 0.2509 | 0.813 |
| FA | Cingulum (hippocampal) L | NW vs OB | -0.36 | 0.2117 | 0.807 |
| FA | Cingulum (hippocampal) L | OW vs OB | -0.03 | 0.8629 | 0.954 |
| FA | Fornix / stria terminalis R | NW vs OW | -0.16 | 0.5674 | 0.910 |
| FA | Fornix / stria terminalis R | NW vs OB | +0.04 | 0.9037 | 0.965 |
| FA | Fornix / stria terminalis R | OW vs OB | +0.18 | 0.7110 | 0.938 |
| FA | Fornix / stria terminalis L | NW vs OW | -0.31 | 0.3374 | 0.849 |
| FA | Fornix / stria terminalis L | NW vs OB | -0.13 | 0.5469 | 0.906 |
| FA | Fornix / stria terminalis L | OW vs OB | +0.17 | 0.7385 | 0.938 |
| FA | Superior longitudinal fasciculus R | NW vs OW | -0.32 | 0.3344 | 0.849 |
| FA | Superior longitudinal fasciculus R | NW vs OB | -0.33 | 0.2095 | 0.807 |
| FA | Superior longitudinal fasciculus R | OW vs OB | +0.01 | 0.6902 | 0.938 |
| FA | Superior longitudinal fasciculus L | NW vs OW | -0.68 | 0.0484 | 0.643 |
| FA | Superior longitudinal fasciculus L | NW vs OB | -0.71 | 0.0102 | 0.376 |
| FA | Superior longitudinal fasciculus L | OW vs OB | -0.09 | 0.3435 | 0.853 |
| FA | Superior fronto-occipital fasciculus R | NW vs OW | -0.06 | 0.8397 | 0.952 |
| FA | Superior fronto-occipital fasciculus R | NW vs OB | +0.10 | 0.6886 | 0.938 |
| FA | Superior fronto-occipital fasciculus R | OW vs OB | +0.16 | 0.5630 | 0.910 |
| FA | Superior fronto-occipital fasciculus L | NW vs OW | -0.34 | 0.2755 | 0.813 |
| FA | Superior fronto-occipital fasciculus L | NW vs OB | -0.46 | 0.0980 | 0.673 |
| FA | Superior fronto-occipital fasciculus L | OW vs OB | -0.20 | 0.5795 | 0.919 |
| FA | Uncinate fasciculus R | NW vs OW | +0.29 | 0.4374 | 0.894 |
| FA | Uncinate fasciculus R | NW vs OB | +0.13 | 0.5610 | 0.910 |
| FA | Uncinate fasciculus R | OW vs OB | -0.15 | 0.8546 | 0.952 |
| FA | Uncinate fasciculus L | NW vs OW | -0.11 | 0.7547 | 0.938 |
| FA | Uncinate fasciculus L | NW vs OB | -0.24 | 0.3146 | 0.836 |
| FA | Uncinate fasciculus L | OW vs OB | -0.17 | 0.7998 | 0.940 |
| FA | Tapetum R | NW vs OW | -0.03 | 0.8598 | 0.952 |
| FA | Tapetum R | NW vs OB | +0.39 | 0.2028 | 0.794 |
| FA | Tapetum R | OW vs OB | +0.42 | 0.2727 | 0.813 |
| FA | Tapetum L | NW vs OW | +0.15 | 0.5996 | 0.927 |
| FA | Tapetum L | NW vs OB | -0.02 | 0.8532 | 0.952 |
| FA | Tapetum L | OW vs OB | -0.18 | 0.5191 | 0.906 |
| MD | Middle cerebellar peduncle | NW vs OW | +0.35 | 0.1506 | 0.753 |
| MD | Middle cerebellar peduncle | NW vs OB | +0.74 | 0.0165 | 0.432 |
| MD | Middle cerebellar peduncle | OW vs OB | +0.37 | 0.6453 | 0.938 |
| MD | Pontine crossing tract | NW vs OW | +0.33 | 0.1517 | 0.753 |
| MD | Pontine crossing tract | NW vs OB | +0.27 | 0.6101 | 0.927 |
| MD | Pontine crossing tract | OW vs OB | -0.13 | 0.2978 | 0.832 |
| MD | Genu of corpus callosum | NW vs OW | +0.25 | 0.5655 | 0.910 |
| MD | Genu of corpus callosum | NW vs OB | -0.11 | 0.6549 | 0.938 |
| MD | Genu of corpus callosum | OW vs OB | -0.41 | 0.2228 | 0.813 |
| MD | Body of corpus callosum | NW vs OW | +0.34 | 0.4028 | 0.892 |
| MD | Body of corpus callosum | NW vs OB | +0.25 | 0.3470 | 0.854 |
| MD | Body of corpus callosum | OW vs OB | -0.07 | 0.9833 | 0.993 |
| MD | Splenium of corpus callosum | NW vs OW | +0.36 | 0.3323 | 0.849 |
| MD | Splenium of corpus callosum | NW vs OB | +0.83 | 0.0050 | 0.244 |
| MD | Splenium of corpus callosum | OW vs OB | +0.34 | 0.1725 | 0.767 |
| MD | Fornix | NW vs OW | +0.65 | 0.0701 | 0.659 |
| MD | Fornix | NW vs OB | +0.13 | 0.7602 | 0.938 |
| MD | Fornix | OW vs OB | -0.42 | 0.1790 | 0.775 |
| MD | Corticospinal tract R | NW vs OW | -0.14 | 0.7464 | 0.938 |
| MD | Corticospinal tract R | NW vs OB | +0.12 | 0.9934 | 0.997 |
| MD | Corticospinal tract R | OW vs OB | +0.24 | 0.9862 | 0.993 |
| MD | Corticospinal tract L | NW vs OW | -0.43 | 0.1492 | 0.753 |
| MD | Corticospinal tract L | NW vs OB | -0.12 | 0.3813 | 0.879 |
| MD | Corticospinal tract L | OW vs OB | +0.25 | 0.7851 | 0.938 |
| MD | Medial lemniscus R | NW vs OW | +0.18 | 0.4155 | 0.892 |
| MD | Medial lemniscus R | NW vs OB | +0.08 | 0.7328 | 0.938 |
| MD | Medial lemniscus R | OW vs OB | -0.08 | 0.8913 | 0.963 |
| MD | Medial lemniscus L | NW vs OW | -0.17 | 0.9793 | 0.991 |
| MD | Medial lemniscus L | NW vs OB | -0.27 | 0.3856 | 0.879 |
| MD | Medial lemniscus L | OW vs OB | -0.11 | 0.8488 | 0.952 |
| MD | Inferior cerebellar peduncle R | NW vs OW | +0.18 | 0.5755 | 0.916 |
| MD | Inferior cerebellar peduncle R | NW vs OB | +0.99 | 0.0025 | 0.211 |
| MD | Inferior cerebellar peduncle R | OW vs OB | +0.81 | 0.0073 | 0.325 |
| MD | Inferior cerebellar peduncle L | NW vs OW | +0.44 | 0.2131 | 0.807 |
| MD | Inferior cerebellar peduncle L | NW vs OB | +0.15 | 0.8727 | 0.957 |
| MD | Inferior cerebellar peduncle L | OW vs OB | -0.27 | 0.2448 | 0.813 |
| MD | Superior cerebellar peduncle R | NW vs OW | +0.50 | 0.0792 | 0.659 |
| MD | Superior cerebellar peduncle R | NW vs OB | +0.48 | 0.2000 | 0.794 |
| MD | Superior cerebellar peduncle R | OW vs OB | -0.05 | 0.5113 | 0.906 |
| MD | Superior cerebellar peduncle L | NW vs OW | +0.23 | 0.2347 | 0.813 |
| MD | Superior cerebellar peduncle L | NW vs OB | +0.51 | 0.0861 | 0.659 |
| MD | Superior cerebellar peduncle L | OW vs OB | +0.29 | 0.6798 | 0.938 |
| MD | Cerebral peduncle R | NW vs OW | -0.06 | 0.8860 | 0.963 |
| MD | Cerebral peduncle R | NW vs OB | +0.10 | 0.7555 | 0.938 |
| MD | Cerebral peduncle R | OW vs OB | +0.17 | 0.7757 | 0.938 |
| MD | Cerebral peduncle L | NW vs OW | -0.13 | 0.7159 | 0.938 |
| MD | Cerebral peduncle L | NW vs OB | -0.23 | 0.4121 | 0.892 |
| MD | Cerebral peduncle L | OW vs OB | -0.11 | 0.5271 | 0.906 |
| MD | Anterior limb of internal capsule R | NW vs OW | +0.27 | 0.6049 | 0.927 |
| MD | Anterior limb of internal capsule R | NW vs OB | +0.01 | 0.7115 | 0.938 |
| MD | Anterior limb of internal capsule R | OW vs OB | -0.29 | 0.8295 | 0.952 |
| MD | Anterior limb of internal capsule L | NW vs OW | +0.26 | 0.7720 | 0.938 |
| MD | Anterior limb of internal capsule L | NW vs OB | +0.19 | 0.4505 | 0.901 |
| MD | Anterior limb of internal capsule L | OW vs OB | -0.13 | 0.4216 | 0.892 |
| MD | Posterior limb of internal capsule R | NW vs OW | +0.68 | 0.0676 | 0.659 |
| MD | Posterior limb of internal capsule R | NW vs OB | +0.29 | 0.1749 | 0.767 |
| MD | Posterior limb of internal capsule R | OW vs OB | -0.43 | 0.5673 | 0.910 |
| MD | Posterior limb of internal capsule L | NW vs OW | +0.20 | 0.8018 | 0.941 |
| MD | Posterior limb of internal capsule L | NW vs OB | +0.07 | 0.6022 | 0.927 |
| MD | Posterior limb of internal capsule L | OW vs OB | -0.15 | 0.8533 | 0.952 |
| MD | Retrolenticular internal capsule R | NW vs OW | +0.44 | 0.2183 | 0.811 |
| MD | Retrolenticular internal capsule R | NW vs OB | +0.13 | 0.4253 | 0.892 |
| MD | Retrolenticular internal capsule R | OW vs OB | -0.33 | 0.4563 | 0.905 |
| MD | Retrolenticular internal capsule L | NW vs OW | +0.30 | 0.5558 | 0.906 |
| MD | Retrolenticular internal capsule L | NW vs OB | +0.05 | 0.7213 | 0.938 |
| MD | Retrolenticular internal capsule L | OW vs OB | -0.26 | 0.7420 | 0.938 |
| MD | Anterior corona radiata R | NW vs OW | +0.23 | 0.8223 | 0.950 |
| MD | Anterior corona radiata R | NW vs OB | +0.03 | 0.7318 | 0.938 |
| MD | Anterior corona radiata R | OW vs OB | -0.20 | 0.9784 | 0.991 |
| MD | Anterior corona radiata L | NW vs OW | +0.34 | 0.5129 | 0.906 |
| MD | Anterior corona radiata L | NW vs OB | +0.03 | 0.7293 | 0.938 |
| MD | Anterior corona radiata L | OW vs OB | -0.32 | 0.6411 | 0.938 |
| MD | Superior corona radiata R | NW vs OW | +0.53 | 0.1880 | 0.793 |
| MD | Superior corona radiata R | NW vs OB | +0.25 | 0.2780 | 0.813 |
| MD | Superior corona radiata R | OW vs OB | -0.30 | 0.7972 | 0.940 |
| MD | Superior corona radiata L | NW vs OW | +0.57 | 0.1512 | 0.753 |
| MD | Superior corona radiata L | NW vs OB | +0.49 | 0.0564 | 0.650 |
| MD | Superior corona radiata L | OW vs OB | -0.12 | 0.7918 | 0.939 |
| MD | Posterior corona radiata R | NW vs OW | +0.24 | 0.4973 | 0.906 |
| MD | Posterior corona radiata R | NW vs OB | +0.41 | 0.0916 | 0.664 |
| MD | Posterior corona radiata R | OW vs OB | +0.08 | 0.6011 | 0.927 |
| MD | Posterior corona radiata L | NW vs OW | +0.25 | 0.5164 | 0.906 |
| MD | Posterior corona radiata L | NW vs OB | +0.40 | 0.1189 | 0.702 |
| MD | Posterior corona radiata L | OW vs OB | +0.07 | 0.7060 | 0.938 |
| MD | Posterior thalamic radiation R | NW vs OW | +0.20 | 0.5491 | 0.906 |
| MD | Posterior thalamic radiation R | NW vs OB | +0.05 | 0.8172 | 0.949 |
| MD | Posterior thalamic radiation R | OW vs OB | -0.18 | 0.4339 | 0.894 |
| MD | Posterior thalamic radiation L | NW vs OW | +0.38 | 0.2835 | 0.813 |
| MD | Posterior thalamic radiation L | NW vs OB | +0.29 | 0.1955 | 0.793 |
| MD | Posterior thalamic radiation L | OW vs OB | -0.12 | 0.6714 | 0.938 |
| MD | Sagittal stratum R | NW vs OW | +0.44 | 0.2331 | 0.813 |
| MD | Sagittal stratum R | NW vs OB | +0.06 | 0.7722 | 0.938 |
| MD | Sagittal stratum R | OW vs OB | -0.43 | 0.1575 | 0.758 |
| MD | Sagittal stratum L | NW vs OW | +0.60 | 0.0551 | 0.648 |
| MD | Sagittal stratum L | NW vs OB | +0.42 | 0.1130 | 0.702 |
| MD | Sagittal stratum L | OW vs OB | -0.22 | 0.4100 | 0.892 |
| MD | External capsule R | NW vs OW | +0.58 | 0.1437 | 0.753 |
| MD | External capsule R | NW vs OB | +0.27 | 0.3165 | 0.836 |
| MD | External capsule R | OW vs OB | -0.39 | 0.7170 | 0.938 |
| MD | External capsule L | NW vs OW | +0.32 | 0.6578 | 0.938 |
| MD | External capsule L | NW vs OB | +0.19 | 0.5570 | 0.906 |
| MD | External capsule L | OW vs OB | -0.16 | 0.6726 | 0.938 |
| MD | Cingulum (cingulate gyrus) R | NW vs OW | +0.35 | 0.2316 | 0.813 |
| MD | Cingulum (cingulate gyrus) R | NW vs OB | +0.27 | 0.5517 | 0.906 |
| MD | Cingulum (cingulate gyrus) R | OW vs OB | -0.13 | 0.5898 | 0.921 |
| MD | Cingulum (cingulate gyrus) L | NW vs OW | +0.40 | 0.1755 | 0.767 |
| MD | Cingulum (cingulate gyrus) L | NW vs OB | +0.62 | 0.0345 | 0.598 |
| MD | Cingulum (cingulate gyrus) L | OW vs OB | +0.06 | 0.8186 | 0.949 |
| MD | Cingulum (hippocampal) R | NW vs OW | +0.25 | 0.3567 | 0.855 |
| MD | Cingulum (hippocampal) R | NW vs OB | +0.68 | 0.0113 | 0.383 |
| MD | Cingulum (hippocampal) R | OW vs OB | +0.31 | 0.2477 | 0.813 |
| MD | Cingulum (hippocampal) L | NW vs OW | +0.04 | 0.7302 | 0.938 |
| MD | Cingulum (hippocampal) L | NW vs OB | +0.19 | 0.3992 | 0.891 |
| MD | Cingulum (hippocampal) L | OW vs OB | +0.12 | 0.9467 | 0.981 |
| MD | Fornix / stria terminalis R | NW vs OW | +0.35 | 0.2734 | 0.813 |
| MD | Fornix / stria terminalis R | NW vs OB | +0.11 | 0.7190 | 0.938 |
| MD | Fornix / stria terminalis R | OW vs OB | -0.23 | 0.5859 | 0.919 |
| MD | Fornix / stria terminalis L | NW vs OW | +0.10 | 0.6788 | 0.938 |
| MD | Fornix / stria terminalis L | NW vs OB | -0.02 | 0.8673 | 0.957 |
| MD | Fornix / stria terminalis L | OW vs OB | -0.10 | 0.9936 | 0.997 |
| MD | Superior longitudinal fasciculus R | NW vs OW | +0.35 | 0.4923 | 0.906 |
| MD | Superior longitudinal fasciculus R | NW vs OB | +0.21 | 0.4008 | 0.891 |
| MD | Superior longitudinal fasciculus R | OW vs OB | -0.15 | 0.8784 | 0.960 |
| MD | Superior longitudinal fasciculus L | NW vs OW | +0.34 | 0.5541 | 0.906 |
| MD | Superior longitudinal fasciculus L | NW vs OB | +0.24 | 0.3047 | 0.836 |
| MD | Superior longitudinal fasciculus L | OW vs OB | -0.12 | 0.9513 | 0.984 |
| MD | Superior fronto-occipital fasciculus R | NW vs OW | -0.11 | 0.3852 | 0.879 |
| MD | Superior fronto-occipital fasciculus R | NW vs OB | -0.37 | 0.3047 | 0.836 |
| MD | Superior fronto-occipital fasciculus R | OW vs OB | -0.30 | 0.9458 | 0.981 |
| MD | Superior fronto-occipital fasciculus L | NW vs OW | +0.14 | 0.8459 | 0.952 |
| MD | Superior fronto-occipital fasciculus L | NW vs OB | +0.05 | 0.7528 | 0.938 |
| MD | Superior fronto-occipital fasciculus L | OW vs OB | -0.13 | 0.6678 | 0.938 |
| MD | Uncinate fasciculus R | NW vs OW | +0.67 | 0.0043 | 0.244 |
| MD | Uncinate fasciculus R | NW vs OB | -0.00 | 0.8508 | 0.952 |
| MD | Uncinate fasciculus R | OW vs OB | -0.52 | 0.0126 | 0.402 |
| MD | Uncinate fasciculus L | NW vs OW | +0.08 | 0.9623 | 0.990 |
| MD | Uncinate fasciculus L | NW vs OB | +0.22 | 0.4587 | 0.905 |
| MD | Uncinate fasciculus L | OW vs OB | +0.10 | 0.3561 | 0.855 |
| MD | Tapetum R | NW vs OW | +0.21 | 0.5849 | 0.919 |
| MD | Tapetum R | NW vs OB | -0.53 | 0.0762 | 0.659 |
| MD | Tapetum R | OW vs OB | -0.69 | 0.0655 | 0.659 |
| MD | Tapetum L | NW vs OW | -0.19 | 0.5959 | 0.927 |
| MD | Tapetum L | NW vs OB | -0.07 | 0.8735 | 0.957 |
| MD | Tapetum L | OW vs OB | +0.13 | 0.6974 | 0.938 |
| AD | Middle cerebellar peduncle | NW vs OW | +0.31 | 0.2645 | 0.813 |
| AD | Middle cerebellar peduncle | NW vs OB | +0.72 | 0.0210 | 0.504 |
| AD | Middle cerebellar peduncle | OW vs OB | +0.38 | 0.5261 | 0.906 |
| AD | Pontine crossing tract | NW vs OW | +0.10 | 0.4952 | 0.906 |
| AD | Pontine crossing tract | NW vs OB | -0.13 | 0.4448 | 0.897 |
| AD | Pontine crossing tract | OW vs OB | -0.15 | 0.3941 | 0.887 |
| AD | Genu of corpus callosum | NW vs OW | +0.23 | 0.5122 | 0.906 |
| AD | Genu of corpus callosum | NW vs OB | -0.04 | 0.9406 | 0.981 |
| AD | Genu of corpus callosum | OW vs OB | -0.37 | 0.2314 | 0.813 |
| AD | Body of corpus callosum | NW vs OW | -0.04 | 0.7828 | 0.938 |
| AD | Body of corpus callosum | NW vs OB | -0.28 | 0.2770 | 0.813 |
| AD | Body of corpus callosum | OW vs OB | -0.20 | 0.5400 | 0.906 |
| AD | Splenium of corpus callosum | NW vs OW | +0.28 | 0.4203 | 0.892 |
| AD | Splenium of corpus callosum | NW vs OB | +0.45 | 0.2023 | 0.794 |
| AD | Splenium of corpus callosum | OW vs OB | +0.13 | 0.7281 | 0.938 |
| AD | Fornix | NW vs OW | +0.46 | 0.2786 | 0.813 |
| AD | Fornix | NW vs OB | +0.13 | 0.7207 | 0.938 |
| AD | Fornix | OW vs OB | -0.29 | 0.5546 | 0.906 |
| AD | Corticospinal tract R | NW vs OW | -0.07 | 0.9611 | 0.990 |
| AD | Corticospinal tract R | NW vs OB | +0.19 | 0.7678 | 0.938 |
| AD | Corticospinal tract R | OW vs OB | +0.24 | 0.9719 | 0.990 |
| AD | Corticospinal tract L | NW vs OW | -0.26 | 0.4157 | 0.892 |
| AD | Corticospinal tract L | NW vs OB | +0.05 | 0.8598 | 0.952 |
| AD | Corticospinal tract L | OW vs OB | +0.31 | 0.9951 | 0.997 |
| AD | Medial lemniscus R | NW vs OW | +0.26 | 0.2675 | 0.813 |
| AD | Medial lemniscus R | NW vs OB | +0.15 | 0.5081 | 0.906 |
| AD | Medial lemniscus R | OW vs OB | -0.07 | 0.9753 | 0.991 |
| AD | Medial lemniscus L | NW vs OW | +0.05 | 0.5900 | 0.921 |
| AD | Medial lemniscus L | NW vs OB | -0.11 | 0.8882 | 0.963 |
| AD | Medial lemniscus L | OW vs OB | -0.16 | 0.6366 | 0.938 |
| AD | Inferior cerebellar peduncle R | NW vs OW | -0.01 | 0.9102 | 0.966 |
| AD | Inferior cerebellar peduncle R | NW vs OB | +0.59 | 0.0831 | 0.659 |
| AD | Inferior cerebellar peduncle R | OW vs OB | +0.57 | 0.0874 | 0.659 |
| AD | Inferior cerebellar peduncle L | NW vs OW | +0.45 | 0.1806 | 0.776 |
| AD | Inferior cerebellar peduncle L | NW vs OB | -0.12 | 0.4410 | 0.894 |
| AD | Inferior cerebellar peduncle L | OW vs OB | -0.58 | 0.0353 | 0.598 |
| AD | Superior cerebellar peduncle R | NW vs OW | +0.51 | 0.1347 | 0.725 |
| AD | Superior cerebellar peduncle R | NW vs OB | +0.39 | 0.3107 | 0.836 |
| AD | Superior cerebellar peduncle R | OW vs OB | -0.15 | 0.5223 | 0.906 |
| AD | Superior cerebellar peduncle L | NW vs OW | +0.34 | 0.2156 | 0.811 |
| AD | Superior cerebellar peduncle L | NW vs OB | +0.50 | 0.0807 | 0.659 |
| AD | Superior cerebellar peduncle L | OW vs OB | +0.16 | 0.7068 | 0.938 |
| AD | Cerebral peduncle R | NW vs OW | +0.17 | 0.2571 | 0.813 |
| AD | Cerebral peduncle R | NW vs OB | +0.60 | 0.0654 | 0.659 |
| AD | Cerebral peduncle R | OW vs OB | +0.27 | 0.8992 | 0.965 |
| AD | Cerebral peduncle L | NW vs OW | -0.17 | 0.7232 | 0.938 |
| AD | Cerebral peduncle L | NW vs OB | +0.26 | 0.4129 | 0.892 |
| AD | Cerebral peduncle L | OW vs OB | +0.40 | 0.4327 | 0.894 |
| AD | Anterior limb of internal capsule R | NW vs OW | -0.12 | 0.5074 | 0.906 |
| AD | Anterior limb of internal capsule R | NW vs OB | +0.50 | 0.0965 | 0.673 |
| AD | Anterior limb of internal capsule R | OW vs OB | +0.57 | 0.0274 | 0.598 |
| AD | Anterior limb of internal capsule L | NW vs OW | +0.04 | 0.6583 | 0.938 |
| AD | Anterior limb of internal capsule L | NW vs OB | +0.47 | 0.1174 | 0.702 |
| AD | Anterior limb of internal capsule L | OW vs OB | +0.45 | 0.0328 | 0.598 |
| AD | Posterior limb of internal capsule R | NW vs OW | +0.39 | 0.1905 | 0.793 |
| AD | Posterior limb of internal capsule R | NW vs OB | +0.44 | 0.1107 | 0.702 |
| AD | Posterior limb of internal capsule R | OW vs OB | -0.02 | 0.9231 | 0.969 |
| AD | Posterior limb of internal capsule L | NW vs OW | +0.11 | 0.8734 | 0.957 |
| AD | Posterior limb of internal capsule L | NW vs OB | +0.11 | 0.5334 | 0.906 |
| AD | Posterior limb of internal capsule L | OW vs OB | +0.00 | 0.7393 | 0.938 |
| AD | Retrolenticular internal capsule R | NW vs OW | +0.19 | 0.6523 | 0.938 |
| AD | Retrolenticular internal capsule R | NW vs OB | +0.09 | 0.5375 | 0.906 |
| AD | Retrolenticular internal capsule R | OW vs OB | -0.13 | 0.9273 | 0.971 |
| AD | Retrolenticular internal capsule L | NW vs OW | +0.22 | 0.9017 | 0.965 |
| AD | Retrolenticular internal capsule L | NW vs OB | +0.21 | 0.2633 | 0.813 |
| AD | Retrolenticular internal capsule L | OW vs OB | -0.04 | 0.7359 | 0.938 |
| AD | Anterior corona radiata R | NW vs OW | +0.10 | 0.9859 | 0.993 |
| AD | Anterior corona radiata R | NW vs OB | -0.31 | 0.4171 | 0.892 |
| AD | Anterior corona radiata R | OW vs OB | -0.44 | 0.3580 | 0.855 |
| AD | Anterior corona radiata L | NW vs OW | +0.12 | 0.9174 | 0.966 |
| AD | Anterior corona radiata L | NW vs OB | -0.20 | 0.7620 | 0.938 |
| AD | Anterior corona radiata L | OW vs OB | -0.32 | 0.4470 | 0.897 |
| AD | Superior corona radiata R | NW vs OW | +0.09 | 0.9359 | 0.978 |
| AD | Superior corona radiata R | NW vs OB | +0.04 | 0.9061 | 0.965 |
| AD | Superior corona radiata R | OW vs OB | -0.05 | 0.7096 | 0.938 |
| AD | Superior corona radiata L | NW vs OW | +0.19 | 0.7850 | 0.938 |
| AD | Superior corona radiata L | NW vs OB | +0.28 | 0.3837 | 0.879 |
| AD | Superior corona radiata L | OW vs OB | +0.06 | 0.7517 | 0.938 |
| AD | Posterior corona radiata R | NW vs OW | +0.11 | 0.9050 | 0.965 |
| AD | Posterior corona radiata R | NW vs OB | +0.34 | 0.2792 | 0.813 |
| AD | Posterior corona radiata R | OW vs OB | +0.15 | 0.6268 | 0.938 |
| AD | Posterior corona radiata L | NW vs OW | +0.18 | 0.7464 | 0.938 |
| AD | Posterior corona radiata L | NW vs OB | +0.26 | 0.4204 | 0.892 |
| AD | Posterior corona radiata L | OW vs OB | +0.03 | 0.8430 | 0.952 |
| AD | Posterior thalamic radiation R | NW vs OW | +0.01 | 0.9440 | 0.981 |
| AD | Posterior thalamic radiation R | NW vs OB | -0.21 | 0.4700 | 0.906 |
| AD | Posterior thalamic radiation R | OW vs OB | -0.26 | 0.2988 | 0.832 |
| AD | Posterior thalamic radiation L | NW vs OW | +0.23 | 0.4140 | 0.892 |
| AD | Posterior thalamic radiation L | NW vs OB | +0.11 | 0.4676 | 0.906 |
| AD | Posterior thalamic radiation L | OW vs OB | -0.12 | 0.6127 | 0.929 |
| AD | Sagittal stratum R | NW vs OW | -0.01 | 0.8553 | 0.952 |
| AD | Sagittal stratum R | NW vs OB | -0.12 | 0.7204 | 0.938 |
| AD | Sagittal stratum R | OW vs OB | -0.14 | 0.5361 | 0.906 |
| AD | Sagittal stratum L | NW vs OW | +0.25 | 0.3781 | 0.879 |
| AD | Sagittal stratum L | NW vs OB | +0.33 | 0.2229 | 0.813 |
| AD | Sagittal stratum L | OW vs OB | +0.04 | 0.8350 | 0.952 |
| AD | External capsule R | NW vs OW | +0.49 | 0.3016 | 0.835 |
| AD | External capsule R | NW vs OB | +0.48 | 0.0637 | 0.659 |
| AD | External capsule R | OW vs OB | -0.04 | 0.1928 | 0.793 |
| AD | External capsule L | NW vs OW | +0.23 | 0.8575 | 0.952 |
| AD | External capsule L | NW vs OB | +0.40 | 0.1579 | 0.758 |
| AD | External capsule L | OW vs OB | +0.17 | 0.1206 | 0.702 |
| AD | Cingulum (cingulate gyrus) R | NW vs OW | -0.14 | 0.5142 | 0.906 |
| AD | Cingulum (cingulate gyrus) R | NW vs OB | -0.63 | 0.0298 | 0.598 |
| AD | Cingulum (cingulate gyrus) R | OW vs OB | -0.51 | 0.2838 | 0.813 |
| AD | Cingulum (cingulate gyrus) L | NW vs OW | -0.25 | 0.5113 | 0.906 |
| AD | Cingulum (cingulate gyrus) L | NW vs OB | -0.65 | 0.0429 | 0.643 |
| AD | Cingulum (cingulate gyrus) L | OW vs OB | -0.33 | 0.4471 | 0.897 |
| AD | Cingulum (hippocampal) R | NW vs OW | -0.20 | 0.6678 | 0.938 |
| AD | Cingulum (hippocampal) R | NW vs OB | -0.29 | 0.3526 | 0.855 |
| AD | Cingulum (hippocampal) R | OW vs OB | -0.10 | 0.3102 | 0.836 |
| AD | Cingulum (hippocampal) L | NW vs OW | -0.33 | 0.4173 | 0.892 |
| AD | Cingulum (hippocampal) L | NW vs OB | -0.31 | 0.3521 | 0.855 |
| AD | Cingulum (hippocampal) L | OW vs OB | -0.03 | 0.6454 | 0.938 |
| AD | Fornix / stria terminalis R | NW vs OW | +0.35 | 0.2543 | 0.813 |
| AD | Fornix / stria terminalis R | NW vs OB | +0.14 | 0.6419 | 0.938 |
| AD | Fornix / stria terminalis R | OW vs OB | -0.23 | 0.5218 | 0.906 |
| AD | Fornix / stria terminalis L | NW vs OW | -0.19 | 0.6397 | 0.938 |
| AD | Fornix / stria terminalis L | NW vs OB | -0.19 | 0.6876 | 0.938 |
| AD | Fornix / stria terminalis L | OW vs OB | -0.01 | 0.7885 | 0.938 |
| AD | Superior longitudinal fasciculus R | NW vs OW | +0.10 | 0.9692 | 0.990 |
| AD | Superior longitudinal fasciculus R | NW vs OB | -0.18 | 0.5128 | 0.906 |
| AD | Superior longitudinal fasciculus R | OW vs OB | -0.24 | 0.4407 | 0.894 |
| AD | Superior longitudinal fasciculus L | NW vs OW | -0.07 | 0.4290 | 0.892 |
| AD | Superior longitudinal fasciculus L | NW vs OB | -0.47 | 0.0991 | 0.673 |
| AD | Superior longitudinal fasciculus L | OW vs OB | -0.33 | 0.3414 | 0.851 |
| AD | Superior fronto-occipital fasciculus R | NW vs OW | -0.15 | 0.4845 | 0.906 |
| AD | Superior fronto-occipital fasciculus R | NW vs OB | -0.27 | 0.5819 | 0.919 |
| AD | Superior fronto-occipital fasciculus R | OW vs OB | -0.13 | 0.6161 | 0.929 |
| AD | Superior fronto-occipital fasciculus L | NW vs OW | -0.11 | 0.2661 | 0.813 |
| AD | Superior fronto-occipital fasciculus L | NW vs OB | -0.37 | 0.1733 | 0.767 |
| AD | Superior fronto-occipital fasciculus L | OW vs OB | -0.30 | 0.7434 | 0.938 |
| AD | Uncinate fasciculus R | NW vs OW | +0.69 | 0.0163 | 0.432 |
| AD | Uncinate fasciculus R | NW vs OB | +0.10 | 0.7040 | 0.938 |
| AD | Uncinate fasciculus R | OW vs OB | -0.51 | 0.1194 | 0.702 |
| AD | Uncinate fasciculus L | NW vs OW | -0.02 | 0.8546 | 0.952 |
| AD | Uncinate fasciculus L | NW vs OB | -0.11 | 0.5166 | 0.906 |
| AD | Uncinate fasciculus L | OW vs OB | -0.09 | 0.7974 | 0.940 |
| AD | Tapetum R | NW vs OW | +0.23 | 0.4691 | 0.906 |
| AD | Tapetum R | NW vs OB | -0.60 | 0.0459 | 0.643 |
| AD | Tapetum R | OW vs OB | -0.76 | 0.0414 | 0.643 |
| AD | Tapetum L | NW vs OW | -0.24 | 0.5232 | 0.906 |
| AD | Tapetum L | NW vs OB | -0.19 | 0.5693 | 0.911 |
| AD | Tapetum L | OW vs OB | +0.06 | 0.9193 | 0.966 |
| RD | Middle cerebellar peduncle | NW vs OW | +0.37 | 0.1170 | 0.702 |
| RD | Middle cerebellar peduncle | NW vs OB | +0.74 | 0.0163 | 0.432 |
| RD | Middle cerebellar peduncle | OW vs OB | +0.36 | 0.7115 | 0.938 |
| RD | Pontine crossing tract | NW vs OW | +0.42 | 0.0922 | 0.664 |
| RD | Pontine crossing tract | NW vs OB | +0.36 | 0.3814 | 0.879 |
| RD | Pontine crossing tract | OW vs OB | -0.11 | 0.3081 | 0.836 |
| RD | Genu of corpus callosum | NW vs OW | +0.24 | 0.6102 | 0.927 |
| RD | Genu of corpus callosum | NW vs OB | -0.13 | 0.5499 | 0.906 |
| RD | Genu of corpus callosum | OW vs OB | -0.41 | 0.2429 | 0.813 |
| RD | Body of corpus callosum | NW vs OW | +0.45 | 0.2197 | 0.811 |
| RD | Body of corpus callosum | NW vs OB | +0.55 | 0.0474 | 0.643 |
| RD | Body of corpus callosum | OW vs OB | +0.04 | 0.6358 | 0.938 |
| RD | Splenium of corpus callosum | NW vs OW | +0.34 | 0.3973 | 0.890 |
| RD | Splenium of corpus callosum | NW vs OB | +0.90 | 0.0014 | 0.161 |
| RD | Splenium of corpus callosum | OW vs OB | +0.41 | 0.0679 | 0.659 |
| RD | Fornix | NW vs OW | +0.68 | 0.0481 | 0.643 |
| RD | Fornix | NW vs OB | +0.12 | 0.7816 | 0.938 |
| RD | Fornix | OW vs OB | -0.45 | 0.1132 | 0.702 |
| RD | Corticospinal tract R | NW vs OW | -0.17 | 0.6545 | 0.938 |
| RD | Corticospinal tract R | NW vs OB | +0.09 | 0.9047 | 0.965 |
| RD | Corticospinal tract R | OW vs OB | +0.24 | 0.9703 | 0.990 |
| RD | Corticospinal tract L | NW vs OW | -0.49 | 0.0881 | 0.659 |
| RD | Corticospinal tract L | NW vs OB | -0.20 | 0.2354 | 0.813 |
| RD | Corticospinal tract L | OW vs OB | +0.20 | 0.6937 | 0.938 |
| RD | Medial lemniscus R | NW vs OW | +0.10 | 0.6458 | 0.938 |
| RD | Medial lemniscus R | NW vs OB | +0.02 | 0.9428 | 0.981 |
| RD | Medial lemniscus R | OW vs OB | -0.07 | 0.8148 | 0.949 |
| RD | Medial lemniscus L | NW vs OW | -0.27 | 0.6506 | 0.938 |
| RD | Medial lemniscus L | NW vs OB | -0.29 | 0.2781 | 0.813 |
| RD | Medial lemniscus L | OW vs OB | -0.03 | 0.8946 | 0.963 |
| RD | Inferior cerebellar peduncle R | NW vs OW | +0.28 | 0.4615 | 0.906 |
| RD | Inferior cerebellar peduncle R | NW vs OB | +1.10 | 0.0007 | 0.161 |
| RD | Inferior cerebellar peduncle R | OW vs OB | +0.84 | 0.0051 | 0.244 |
| RD | Inferior cerebellar peduncle L | NW vs OW | +0.36 | 0.3185 | 0.838 |
| RD | Inferior cerebellar peduncle L | NW vs OB | +0.30 | 0.4568 | 0.905 |
| RD | Inferior cerebellar peduncle L | OW vs OB | -0.05 | 0.6984 | 0.938 |
| RD | Superior cerebellar peduncle R | NW vs OW | +0.47 | 0.0701 | 0.659 |
| RD | Superior cerebellar peduncle R | NW vs OB | +0.51 | 0.1718 | 0.767 |
| RD | Superior cerebellar peduncle R | OW vs OB | +0.01 | 0.5215 | 0.906 |
| RD | Superior cerebellar peduncle L | NW vs OW | +0.16 | 0.2776 | 0.813 |
| RD | Superior cerebellar peduncle L | NW vs OB | +0.49 | 0.1020 | 0.683 |
| RD | Superior cerebellar peduncle L | OW vs OB | +0.33 | 0.6857 | 0.938 |
| RD | Cerebral peduncle R | NW vs OW | -0.18 | 0.6907 | 0.938 |
| RD | Cerebral peduncle R | NW vs OB | -0.13 | 0.6904 | 0.938 |
| RD | Cerebral peduncle R | OW vs OB | +0.06 | 0.7634 | 0.938 |
| RD | Cerebral peduncle L | NW vs OW | -0.07 | 0.7859 | 0.938 |
| RD | Cerebral peduncle L | NW vs OB | -0.47 | 0.1220 | 0.703 |
| RD | Cerebral peduncle L | OW vs OB | -0.50 | 0.1279 | 0.715 |
| RD | Anterior limb of internal capsule R | NW vs OW | +0.42 | 0.2759 | 0.813 |
| RD | Anterior limb of internal capsule R | NW vs OB | -0.29 | 0.5408 | 0.906 |
| RD | Anterior limb of internal capsule R | OW vs OB | -0.65 | 0.2411 | 0.813 |
| RD | Anterior limb of internal capsule L | NW vs OW | +0.34 | 0.4838 | 0.906 |
| RD | Anterior limb of internal capsule L | NW vs OB | -0.09 | 0.9153 | 0.966 |
| RD | Anterior limb of internal capsule L | OW vs OB | -0.47 | 0.6259 | 0.938 |
| RD | Posterior limb of internal capsule R | NW vs OW | +0.33 | 0.5367 | 0.906 |
| RD | Posterior limb of internal capsule R | NW vs OB | -0.17 | 0.7234 | 0.938 |
| RD | Posterior limb of internal capsule R | OW vs OB | -0.44 | 0.4960 | 0.906 |
| RD | Posterior limb of internal capsule L | NW vs OW | +0.14 | 0.8843 | 0.963 |
| RD | Posterior limb of internal capsule L | NW vs OB | -0.07 | 0.8284 | 0.952 |
| RD | Posterior limb of internal capsule L | OW vs OB | -0.19 | 0.8923 | 0.963 |
| RD | Retrolenticular internal capsule R | NW vs OW | +0.50 | 0.1529 | 0.753 |
| RD | Retrolenticular internal capsule R | NW vs OB | +0.12 | 0.5211 | 0.906 |
| RD | Retrolenticular internal capsule R | OW vs OB | -0.40 | 0.3274 | 0.849 |
| RD | Retrolenticular internal capsule L | NW vs OW | +0.26 | 0.4586 | 0.905 |
| RD | Retrolenticular internal capsule L | NW vs OB | -0.09 | 0.7299 | 0.938 |
| RD | Retrolenticular internal capsule L | OW vs OB | -0.33 | 0.5058 | 0.906 |
| RD | Anterior corona radiata R | NW vs OW | +0.22 | 0.8111 | 0.948 |
| RD | Anterior corona radiata R | NW vs OB | +0.23 | 0.3594 | 0.855 |
| RD | Anterior corona radiata R | OW vs OB | -0.01 | 0.5330 | 0.906 |
| RD | Anterior corona radiata L | NW vs OW | +0.38 | 0.4347 | 0.894 |
| RD | Anterior corona radiata L | NW vs OB | +0.15 | 0.5545 | 0.906 |
| RD | Anterior corona radiata L | OW vs OB | -0.26 | 0.8736 | 0.957 |
| RD | Superior corona radiata R | NW vs OW | +0.63 | 0.0733 | 0.659 |
| RD | Superior corona radiata R | NW vs OB | +0.28 | 0.2289 | 0.813 |
| RD | Superior corona radiata R | OW vs OB | -0.39 | 0.5144 | 0.906 |
| RD | Superior corona radiata L | NW vs OW | +0.69 | 0.0594 | 0.658 |
| RD | Superior corona radiata L | NW vs OB | +0.47 | 0.0512 | 0.643 |
| RD | Superior corona radiata L | OW vs OB | -0.23 | 0.9148 | 0.966 |
| RD | Posterior corona radiata R | NW vs OW | +0.31 | 0.3396 | 0.851 |
| RD | Posterior corona radiata R | NW vs OB | +0.35 | 0.0994 | 0.673 |
| RD | Posterior corona radiata R | OW vs OB | +0.01 | 0.6852 | 0.938 |
| RD | Posterior corona radiata L | NW vs OW | +0.25 | 0.4704 | 0.906 |
| RD | Posterior corona radiata L | NW vs OB | +0.37 | 0.1122 | 0.702 |
| RD | Posterior corona radiata L | OW vs OB | +0.08 | 0.6796 | 0.938 |
| RD | Posterior thalamic radiation R | NW vs OW | +0.28 | 0.3887 | 0.881 |
| RD | Posterior thalamic radiation R | NW vs OB | +0.23 | 0.3859 | 0.879 |
| RD | Posterior thalamic radiation R | OW vs OB | -0.05 | 0.7796 | 0.938 |
| RD | Posterior thalamic radiation L | NW vs OW | +0.42 | 0.2765 | 0.813 |
| RD | Posterior thalamic radiation L | NW vs OB | +0.34 | 0.1666 | 0.767 |
| RD | Posterior thalamic radiation L | OW vs OB | -0.10 | 0.7990 | 0.940 |
| RD | Sagittal stratum R | NW vs OW | +0.61 | 0.0802 | 0.659 |
| RD | Sagittal stratum R | NW vs OB | +0.16 | 0.5480 | 0.906 |
| RD | Sagittal stratum R | OW vs OB | -0.46 | 0.1628 | 0.767 |
| RD | Sagittal stratum L | NW vs OW | +0.73 | 0.0225 | 0.518 |
| RD | Sagittal stratum L | NW vs OB | +0.39 | 0.1399 | 0.746 |
| RD | Sagittal stratum L | OW vs OB | -0.34 | 0.2964 | 0.832 |
| RD | External capsule R | NW vs OW | +0.51 | 0.1689 | 0.767 |
| RD | External capsule R | NW vs OB | -0.04 | 0.8892 | 0.963 |
| RD | External capsule R | OW vs OB | -0.56 | 0.2089 | 0.807 |
| RD | External capsule L | NW vs OW | +0.28 | 0.6143 | 0.929 |
| RD | External capsule L | NW vs OB | -0.16 | 0.5072 | 0.906 |
| RD | External capsule L | OW vs OB | -0.52 | 0.2800 | 0.813 |
| RD | Cingulum (cingulate gyrus) R | NW vs OW | +0.35 | 0.2017 | 0.794 |
| RD | Cingulum (cingulate gyrus) R | NW vs OB | +0.64 | 0.0517 | 0.643 |
| RD | Cingulum (cingulate gyrus) R | OW vs OB | +0.19 | 0.8353 | 0.952 |
| RD | Cingulum (cingulate gyrus) L | NW vs OW | +0.47 | 0.1475 | 0.753 |
| RD | Cingulum (cingulate gyrus) L | NW vs OB | +1.03 | 0.0012 | 0.161 |
| RD | Cingulum (cingulate gyrus) L | OW vs OB | +0.28 | 0.4727 | 0.906 |
| RD | Cingulum (hippocampal) R | NW vs OW | +0.35 | 0.2747 | 0.813 |
| RD | Cingulum (hippocampal) R | NW vs OB | +0.95 | 0.0007 | 0.161 |
| RD | Cingulum (hippocampal) R | OW vs OB | +0.37 | 0.0940 | 0.669 |
| RD | Cingulum (hippocampal) L | NW vs OW | +0.24 | 0.3736 | 0.879 |
| RD | Cingulum (hippocampal) L | NW vs OB | +0.42 | 0.1273 | 0.715 |
| RD | Cingulum (hippocampal) L | OW vs OB | +0.14 | 0.7400 | 0.938 |
| RD | Fornix / stria terminalis R | NW vs OW | +0.31 | 0.3356 | 0.849 |
| RD | Fornix / stria terminalis R | NW vs OB | +0.08 | 0.7892 | 0.938 |
| RD | Fornix / stria terminalis R | OW vs OB | -0.21 | 0.6702 | 0.938 |
| RD | Fornix / stria terminalis L | NW vs OW | +0.25 | 0.3818 | 0.879 |
| RD | Fornix / stria terminalis L | NW vs OB | +0.08 | 0.6083 | 0.927 |
| RD | Fornix / stria terminalis L | OW vs OB | -0.14 | 0.8889 | 0.963 |
| RD | Superior longitudinal fasciculus R | NW vs OW | +0.37 | 0.3838 | 0.879 |
| RD | Superior longitudinal fasciculus R | NW vs OB | +0.38 | 0.1525 | 0.753 |
| RD | Superior longitudinal fasciculus R | OW vs OB | -0.02 | 0.6955 | 0.938 |
| RD | Superior longitudinal fasciculus L | NW vs OW | +0.51 | 0.2128 | 0.807 |
| RD | Superior longitudinal fasciculus L | NW vs OB | +0.56 | 0.0310 | 0.598 |
| RD | Superior longitudinal fasciculus L | OW vs OB | +0.05 | 0.3908 | 0.883 |
| RD | Superior fronto-occipital fasciculus R | NW vs OW | -0.07 | 0.4782 | 0.906 |
| RD | Superior fronto-occipital fasciculus R | NW vs OB | -0.31 | 0.3459 | 0.854 |
| RD | Superior fronto-occipital fasciculus R | OW vs OB | -0.28 | 0.6966 | 0.938 |
| RD | Superior fronto-occipital fasciculus L | NW vs OW | +0.23 | 0.7289 | 0.938 |
| RD | Superior fronto-occipital fasciculus L | NW vs OB | +0.29 | 0.2659 | 0.813 |
| RD | Superior fronto-occipital fasciculus L | OW vs OB | +0.04 | 0.5100 | 0.906 |
| RD | Uncinate fasciculus R | NW vs OW | +0.12 | 0.4777 | 0.906 |
| RD | Uncinate fasciculus R | NW vs OB | -0.10 | 0.5839 | 0.919 |
| RD | Uncinate fasciculus R | OW vs OB | -0.18 | 0.1235 | 0.704 |
| RD | Uncinate fasciculus L | NW vs OW | +0.09 | 0.9600 | 0.990 |
| RD | Uncinate fasciculus L | NW vs OB | +0.25 | 0.3162 | 0.836 |
| RD | Uncinate fasciculus L | OW vs OB | +0.18 | 0.4116 | 0.892 |
| RD | Tapetum R | NW vs OW | +0.20 | 0.6490 | 0.938 |
| RD | Tapetum R | NW vs OB | -0.49 | 0.1043 | 0.690 |
| RD | Tapetum R | OW vs OB | -0.65 | 0.0857 | 0.659 |
| RD | Tapetum L | NW vs OW | -0.17 | 0.6341 | 0.938 |
| RD | Tapetum L | NW vs OB | -0.02 | 0.9724 | 0.990 |
| RD | Tapetum L | OW vs OB | +0.15 | 0.6060 | 0.927 |

NW, normal-weight; OW, overweight; OB, obese; FA, fractional anisotropy; MD, mean diffusivity; AD, axial diffusivity; RD, radial diffusivity.

# **Supplementary Table S2. Complete results of the whole-sample regression on BMI**

Linear regression across all 70 participants with BMI entered as a continuous predictor and age, sex, and educational level as covariates, for each of the 48 regions of the JHU ICBM-DTI-81 atlas and each of the four diffusion metrics (192 tests). Because BMI was not used to define groups and every atlas region was tested without prior selection, these estimates are not subject to selection bias. P values are uncorrected; q values are Benjamini–Hochberg adjusted across the 192 tests. Three associations survived correction, all in the right inferior cerebellar peduncle, and are marked in the final column.

| **Metric** | **Tract (JHU)** | **Standardised β** | **P** | **q** | **q < 0.05** |
| --- | --- | --- | --- | --- | --- |
| FA | Middle cerebellar peduncle | -0.216 | 0.0379 | 0.518 |  |
| FA | Pontine crossing tract | -0.165 | 0.1457 | 0.708 |  |
| FA | Genu of corpus callosum | +0.112 | 0.3417 | 0.897 |  |
| FA | Body of corpus callosum | -0.275 | 0.0232 | 0.424 |  |
| FA | Splenium of corpus callosum | -0.201 | 0.0750 | 0.659 |  |
| FA | Fornix | +0.033 | 0.7766 | 0.930 |  |
| FA | Corticospinal tract R | -0.059 | 0.6229 | 0.930 |  |
| FA | Corticospinal tract L | +0.080 | 0.5165 | 0.918 |  |
| FA | Medial lemniscus R | +0.098 | 0.4310 | 0.905 |  |
| FA | Medial lemniscus L | +0.116 | 0.3430 | 0.897 |  |
| FA | Inferior cerebellar peduncle R | -0.461 | 9.6e-5 | 0.009 | yes |
| FA | Inferior cerebellar peduncle L | -0.149 | 0.2313 | 0.871 |  |
| FA | Superior cerebellar peduncle R | -0.178 | 0.1103 | 0.708 |  |
| FA | Superior cerebellar peduncle L | -0.076 | 0.5043 | 0.905 |  |
| FA | Cerebral peduncle R | +0.109 | 0.3743 | 0.897 |  |
| FA | Cerebral peduncle L | +0.213 | 0.0822 | 0.659 |  |
| FA | Anterior limb of internal capsule R | +0.074 | 0.5261 | 0.918 |  |
| FA | Anterior limb of internal capsule L | +0.080 | 0.4944 | 0.905 |  |
| FA | Posterior limb of internal capsule R | +0.036 | 0.7656 | 0.930 |  |
| FA | Posterior limb of internal capsule L | +0.044 | 0.7251 | 0.930 |  |
| FA | Retrolenticular internal capsule R | -0.057 | 0.6403 | 0.930 |  |
| FA | Retrolenticular internal capsule L | +0.085 | 0.4960 | 0.905 |  |
| FA | Anterior corona radiata R | -0.097 | 0.4332 | 0.905 |  |
| FA | Anterior corona radiata L | -0.107 | 0.3786 | 0.897 |  |
| FA | Superior corona radiata R | -0.030 | 0.8083 | 0.930 |  |
| FA | Superior corona radiata L | -0.028 | 0.8193 | 0.930 |  |
| FA | Posterior corona radiata R | -0.019 | 0.8758 | 0.955 |  |
| FA | Posterior corona radiata L | -0.093 | 0.4561 | 0.905 |  |
| FA | Posterior thalamic radiation R | -0.130 | 0.2972 | 0.873 |  |
| FA | Posterior thalamic radiation L | -0.087 | 0.4832 | 0.905 |  |
| FA | Sagittal stratum R | -0.121 | 0.3265 | 0.897 |  |
| FA | Sagittal stratum L | +0.029 | 0.8128 | 0.930 |  |
| FA | External capsule R | +0.147 | 0.2208 | 0.850 |  |
| FA | External capsule L | +0.195 | 0.1041 | 0.708 |  |
| FA | Cingulum (cingulate gyrus) R | -0.214 | 0.0824 | 0.659 |  |
| FA | Cingulum (cingulate gyrus) L | -0.353 | 0.0037 | 0.180 |  |
| FA | Cingulum (hippocampal) R | -0.281 | 0.0202 | 0.424 |  |
| FA | Cingulum (hippocampal) L | -0.179 | 0.1406 | 0.708 |  |
| FA | Fornix / stria terminalis R | -0.032 | 0.7940 | 0.930 |  |
| FA | Fornix / stria terminalis L | -0.012 | 0.9155 | 0.964 |  |
| FA | Superior longitudinal fasciculus R | -0.087 | 0.4624 | 0.905 |  |
| FA | Superior longitudinal fasciculus L | -0.266 | 0.0276 | 0.424 |  |
| FA | Superior fronto-occipital fasciculus R | +0.035 | 0.7796 | 0.930 |  |
| FA | Superior fronto-occipital fasciculus L | -0.137 | 0.2698 | 0.873 |  |
| FA | Uncinate fasciculus R | -0.013 | 0.9133 | 0.964 |  |
| FA | Uncinate fasciculus L | -0.109 | 0.3849 | 0.898 |  |
| FA | Tapetum R | +0.132 | 0.2795 | 0.873 |  |
| FA | Tapetum L | +0.120 | 0.3248 | 0.897 |  |
| MD | Middle cerebellar peduncle | +0.244 | 0.0274 | 0.424 |  |
| MD | Pontine crossing tract | +0.125 | 0.2740 | 0.873 |  |
| MD | Genu of corpus callosum | +0.026 | 0.8328 | 0.930 |  |
| MD | Body of corpus callosum | +0.002 | 0.9832 | 0.983 |  |
| MD | Splenium of corpus callosum | +0.204 | 0.0579 | 0.581 |  |
| MD | Fornix | +0.046 | 0.6897 | 0.930 |  |
| MD | Corticospinal tract R | +0.090 | 0.4327 | 0.905 |  |
| MD | Corticospinal tract L | +0.055 | 0.6298 | 0.930 |  |
| MD | Medial lemniscus R | -0.005 | 0.9690 | 0.974 |  |
| MD | Medial lemniscus L | -0.106 | 0.3929 | 0.898 |  |
| MD | Inferior cerebellar peduncle R | +0.405 | 0.0002 | 0.014 | yes |
| MD | Inferior cerebellar peduncle L | +0.062 | 0.6117 | 0.930 |  |
| MD | Superior cerebellar peduncle R | +0.154 | 0.1949 | 0.814 |  |
| MD | Superior cerebellar peduncle L | +0.137 | 0.2598 | 0.873 |  |
| MD | Cerebral peduncle R | -0.037 | 0.7683 | 0.930 |  |
| MD | Cerebral peduncle L | -0.104 | 0.4068 | 0.905 |  |
| MD | Anterior limb of internal capsule R | +0.101 | 0.3705 | 0.897 |  |
| MD | Anterior limb of internal capsule L | +0.108 | 0.3514 | 0.897 |  |
| MD | Posterior limb of internal capsule R | +0.078 | 0.5010 | 0.905 |  |
| MD | Posterior limb of internal capsule L | +0.029 | 0.8090 | 0.930 |  |
| MD | Retrolenticular internal capsule R | -0.029 | 0.8074 | 0.930 |  |
| MD | Retrolenticular internal capsule L | -0.035 | 0.7665 | 0.930 |  |
| MD | Anterior corona radiata R | -0.036 | 0.7568 | 0.930 |  |
| MD | Anterior corona radiata L | -0.049 | 0.6634 | 0.930 |  |
| MD | Superior corona radiata R | +0.059 | 0.6102 | 0.930 |  |
| MD | Superior corona radiata L | +0.117 | 0.2984 | 0.873 |  |
| MD | Posterior corona radiata R | +0.013 | 0.9143 | 0.964 |  |
| MD | Posterior corona radiata L | +0.036 | 0.7578 | 0.930 |  |
| MD | Posterior thalamic radiation R | -0.021 | 0.8693 | 0.954 |  |
| MD | Posterior thalamic radiation L | +0.037 | 0.7596 | 0.930 |  |
| MD | Sagittal stratum R | -0.026 | 0.8343 | 0.930 |  |
| MD | Sagittal stratum L | +0.071 | 0.5675 | 0.930 |  |
| MD | External capsule R | +0.120 | 0.2955 | 0.873 |  |
| MD | External capsule L | +0.145 | 0.1935 | 0.814 |  |
| MD | Cingulum (cingulate gyrus) R | +0.035 | 0.7746 | 0.930 |  |
| MD | Cingulum (cingulate gyrus) L | +0.153 | 0.2117 | 0.850 |  |
| MD | Cingulum (hippocampal) R | +0.140 | 0.2497 | 0.873 |  |
| MD | Cingulum (hippocampal) L | +0.042 | 0.7338 | 0.930 |  |
| MD | Fornix / stria terminalis R | +0.092 | 0.4413 | 0.905 |  |
| MD | Fornix / stria terminalis L | -0.047 | 0.7025 | 0.930 |  |
| MD | Superior longitudinal fasciculus R | +0.048 | 0.6708 | 0.930 |  |
| MD | Superior longitudinal fasciculus L | +0.048 | 0.6673 | 0.930 |  |
| MD | Superior fronto-occipital fasciculus R | -0.087 | 0.4652 | 0.905 |  |
| MD | Superior fronto-occipital fasciculus L | +0.032 | 0.7843 | 0.930 |  |
| MD | Uncinate fasciculus R | -0.090 | 0.4316 | 0.905 |  |
| MD | Uncinate fasciculus L | +0.111 | 0.3700 | 0.897 |  |
| MD | Tapetum R | -0.184 | 0.1326 | 0.708 |  |
| MD | Tapetum L | -0.166 | 0.1759 | 0.782 |  |
| AD | Middle cerebellar peduncle | +0.221 | 0.0523 | 0.581 |  |
| AD | Pontine crossing tract | +0.011 | 0.9298 | 0.965 |  |
| AD | Genu of corpus callosum | +0.025 | 0.8383 | 0.930 |  |
| AD | Body of corpus callosum | -0.220 | 0.0605 | 0.581 |  |
| AD | Splenium of corpus callosum | +0.067 | 0.5792 | 0.930 |  |
| AD | Fornix | +0.069 | 0.5523 | 0.928 |  |
| AD | Corticospinal tract R | +0.110 | 0.3412 | 0.897 |  |
| AD | Corticospinal tract L | +0.107 | 0.3530 | 0.897 |  |
| AD | Medial lemniscus R | +0.055 | 0.6561 | 0.930 |  |
| AD | Medial lemniscus L | -0.022 | 0.8544 | 0.943 |  |
| AD | Inferior cerebellar peduncle R | +0.245 | 0.0287 | 0.424 |  |
| AD | Inferior cerebellar peduncle L | +0.013 | 0.9184 | 0.964 |  |
| AD | Superior cerebellar peduncle R | +0.121 | 0.3170 | 0.897 |  |
| AD | Superior cerebellar peduncle L | +0.141 | 0.2573 | 0.873 |  |
| AD | Cerebral peduncle R | +0.051 | 0.6607 | 0.930 |  |
| AD | Cerebral peduncle L | +0.066 | 0.5946 | 0.930 |  |
| AD | Anterior limb of internal capsule R | +0.181 | 0.1435 | 0.708 |  |
| AD | Anterior limb of internal capsule L | +0.171 | 0.1436 | 0.708 |  |
| AD | Posterior limb of internal capsule R | +0.052 | 0.6637 | 0.930 |  |
| AD | Posterior limb of internal capsule L | +0.036 | 0.7674 | 0.930 |  |
| AD | Retrolenticular internal capsule R | -0.077 | 0.5307 | 0.918 |  |
| AD | Retrolenticular internal capsule L | +0.043 | 0.7177 | 0.930 |  |
| AD | Anterior corona radiata R | -0.129 | 0.2831 | 0.873 |  |
| AD | Anterior corona radiata L | -0.147 | 0.2213 | 0.850 |  |
| AD | Superior corona radiata R | +0.027 | 0.8228 | 0.930 |  |
| AD | Superior corona radiata L | +0.092 | 0.4391 | 0.905 |  |
| AD | Posterior corona radiata R | +0.007 | 0.9530 | 0.972 |  |
| AD | Posterior corona radiata L | -0.006 | 0.9571 | 0.972 |  |
| AD | Posterior thalamic radiation R | -0.131 | 0.2896 | 0.873 |  |
| AD | Posterior thalamic radiation L | -0.046 | 0.7067 | 0.930 |  |
| AD | Sagittal stratum R | -0.111 | 0.3719 | 0.897 |  |
| AD | Sagittal stratum L | +0.065 | 0.6011 | 0.930 |  |
| AD | External capsule R | +0.175 | 0.1073 | 0.708 |  |
| AD | External capsule L | +0.215 | 0.0547 | 0.581 |  |
| AD | Cingulum (cingulate gyrus) R | -0.247 | 0.0410 | 0.518 |  |
| AD | Cingulum (cingulate gyrus) L | -0.307 | 0.0125 | 0.374 |  |
| AD | Cingulum (hippocampal) R | -0.191 | 0.1218 | 0.708 |  |
| AD | Cingulum (hippocampal) L | -0.190 | 0.1203 | 0.708 |  |
| AD | Fornix / stria terminalis R | +0.073 | 0.5559 | 0.928 |  |
| AD | Fornix / stria terminalis L | -0.094 | 0.4539 | 0.905 |  |
| AD | Superior longitudinal fasciculus R | -0.078 | 0.4997 | 0.905 |  |
| AD | Superior longitudinal fasciculus L | -0.190 | 0.1102 | 0.708 |  |
| AD | Superior fronto-occipital fasciculus R | -0.054 | 0.6485 | 0.930 |  |
| AD | Superior fronto-occipital fasciculus L | -0.095 | 0.3914 | 0.898 |  |
| AD | Uncinate fasciculus R | -0.087 | 0.4790 | 0.905 |  |
| AD | Uncinate fasciculus L | -0.033 | 0.7926 | 0.930 |  |
| AD | Tapetum R | -0.216 | 0.0786 | 0.659 |  |
| AD | Tapetum L | -0.207 | 0.0937 | 0.708 |  |
| RD | Middle cerebellar peduncle | +0.251 | 0.0211 | 0.424 |  |
| RD | Pontine crossing tract | +0.162 | 0.1437 | 0.708 |  |
| RD | Genu of corpus callosum | +0.024 | 0.8382 | 0.930 |  |
| RD | Body of corpus callosum | +0.144 | 0.2142 | 0.850 |  |
| RD | Splenium of corpus callosum | +0.257 | 0.0137 | 0.374 |  |
| RD | Fornix | +0.035 | 0.7572 | 0.930 |  |
| RD | Corticospinal tract R | +0.079 | 0.4924 | 0.905 |  |
| RD | Corticospinal tract L | +0.027 | 0.8140 | 0.930 |  |
| RD | Medial lemniscus R | -0.045 | 0.7196 | 0.930 |  |
| RD | Medial lemniscus L | -0.128 | 0.3001 | 0.873 |  |
| RD | Inferior cerebellar peduncle R | +0.442 | 6.7e-5 | 0.009 | yes |
| RD | Inferior cerebellar peduncle L | +0.084 | 0.4849 | 0.905 |  |
| RD | Superior cerebellar peduncle R | +0.165 | 0.1582 | 0.741 |  |
| RD | Superior cerebellar peduncle L | +0.127 | 0.2879 | 0.873 |  |
| RD | Cerebral peduncle R | -0.076 | 0.5438 | 0.924 |  |
| RD | Cerebral peduncle L | -0.179 | 0.1476 | 0.708 |  |
| RD | Anterior limb of internal capsule R | +0.013 | 0.9095 | 0.964 |  |
| RD | Anterior limb of internal capsule L | +0.026 | 0.8196 | 0.930 |  |
| RD | Posterior limb of internal capsule R | +0.029 | 0.8057 | 0.930 |  |
| RD | Posterior limb of internal capsule L | -0.006 | 0.9627 | 0.973 |  |
| RD | Retrolenticular internal capsule R | +0.016 | 0.8949 | 0.964 |  |
| RD | Retrolenticular internal capsule L | -0.076 | 0.5330 | 0.918 |  |
| RD | Anterior corona radiata R | +0.030 | 0.7997 | 0.930 |  |
| RD | Anterior corona radiata L | +0.016 | 0.8911 | 0.964 |  |
| RD | Superior corona radiata R | +0.053 | 0.6538 | 0.930 |  |
| RD | Superior corona radiata L | +0.103 | 0.3682 | 0.897 |  |
| RD | Posterior corona radiata R | +0.011 | 0.9253 | 0.965 |  |
| RD | Posterior corona radiata L | +0.053 | 0.6666 | 0.930 |  |
| RD | Posterior thalamic radiation R | +0.069 | 0.5843 | 0.930 |  |
| RD | Posterior thalamic radiation L | +0.084 | 0.4839 | 0.905 |  |
| RD | Sagittal stratum R | +0.030 | 0.8103 | 0.930 |  |
| RD | Sagittal stratum L | +0.061 | 0.6215 | 0.930 |  |
| RD | External capsule R | +0.050 | 0.6790 | 0.930 |  |
| RD | External capsule L | -0.009 | 0.9437 | 0.969 |  |
| RD | Cingulum (cingulate gyrus) R | +0.182 | 0.1391 | 0.708 |  |
| RD | Cingulum (cingulate gyrus) L | +0.326 | 0.0071 | 0.273 |  |
| RD | Cingulum (hippocampal) R | +0.245 | 0.0432 | 0.518 |  |
| RD | Cingulum (hippocampal) L | +0.163 | 0.1793 | 0.782 |  |
| RD | Fornix / stria terminalis R | +0.089 | 0.4547 | 0.905 |  |
| RD | Fornix / stria terminalis L | -0.009 | 0.9378 | 0.968 |  |
| RD | Superior longitudinal fasciculus R | +0.110 | 0.3427 | 0.897 |  |
| RD | Superior longitudinal fasciculus L | +0.176 | 0.1239 | 0.708 |  |
| RD | Superior fronto-occipital fasciculus R | -0.076 | 0.5357 | 0.918 |  |
| RD | Superior fronto-occipital fasciculus L | +0.096 | 0.4352 | 0.905 |  |
| RD | Uncinate fasciculus R | -0.027 | 0.8170 | 0.930 |  |
| RD | Uncinate fasciculus L | +0.131 | 0.2881 | 0.873 |  |
| RD | Tapetum R | -0.165 | 0.1755 | 0.782 |  |
| RD | Tapetum L | -0.145 | 0.2363 | 0.873 |  |

Abbreviations as in Supplementary Table S1.

# **Supplementary Table S3. Evidential status of each multimodal correspondence**

Each region in which more than one imaging index was altered is classified by the level at which the correspondence holds. Peak-level indicates that both indices were independently reduced at the voxel level at co-located or near-co-located peaks; atlas-region level indicates that the two peaks fall within the same atlas-defined region but are not co-located; non-independent indicates that the two indices derive from the same underlying data and therefore do not constitute cross-modal corroboration. Peak separations are Euclidean distances between the reported MNI peak coordinates.

| **Region** | **Indices** | **Peak separation** | **Evidential status** |
| --- | --- | --- | --- |
| Right Crus II / lobule VIII (30, −75, −51) | ALFF × CBF | 12.4 mm | Peak-level; both modalities represented by clusters reported independently in Tables 2 and 5 |
| Left cerebellar lobule VIII (−24, −75, −51) | ALFF × CBF | co-located | Peak-level by conjunction (highest minimum t = 4.48); the ALFF cluster at this site (peak t = −5.19 at −27, −75, −48) did not survive cluster-level correction and is not listed in Table 2 |
| Vermal lobule VIII (0, −57, −45) | ALFF × CBF | 32.3 mm | Atlas-region level only; CBF at the ALFF peak t = −0.97, not significant |
| Right Crus I (44, −52, −46) | ALFF × CBF | n/a | Not supported; ALFF at the CBF peak t = +0.63, opposite in direction and not significant |
| Left superior frontal gyrus (−12, 36, 51) | ALFF × ReHo | 4.2 mm | Closest spatial correspondence in the study, but non-independent: ALFF and ReHo derive from the same resting-state data |
| Cingulum bundle | FA × MD × AD × RD; middle cingulate ReHo | n/a (ROI) | Non-independent: the diffusion metrics derive from the same tensor; group differences reached uncorrected P < 0.05 only |
| Right inferior cerebellar peduncle | FA × MD × RD | n/a (ROI) | ROI-level by design; the only tract whose association with BMI survived FDR correction (q = 0.009–0.014, Supplementary Table S2) |

Abbreviations as in Supplementary Table S1; ALFF, amplitude of low-frequency fluctuation; ReHo, regional homogeneity; CBF, cerebral blood flow.
